# Author Correction: Early Triassic super-greenhouse climate driven by vegetation collapse

**DOI:** 10.1038/s41467-025-65816-7

**Published:** 2025-10-29

**Authors:** Zhen Xu, Jianxin Yu, Hongfu Yin, Andrew S. Merdith, Jason Hilton, Bethany J. Allen, Khushboo Gurung, Paul B. Wignall, Alexander M. Dunhill, Jun Shen, David Schwartzman, Yves Goddéris, Yannick Donnadieu, Yuxuan Wang, Yinggang Zhang, Simon W. Poulton, Benjamin J. W. Mills

**Affiliations:** 1 State Key Laboratory of Geomicrobiology and Environmental Changes, School of Earth Sciences, China University of Geosciences, Wuhan, 430074 P.R. China; 2 School of Earth and Environment, University of Leeds, Leeds, UK; 3 School of Physics, Chemistry and Earth Science, University of Adelaide, Adelaide, SA Australia; 4 Birmingham Institute of Forest Research, University of Birmingham, Edgbaston, Birmingham, UK; 5 Department of Biosystems Science and Engineering, ETH Zürich, Basel, Switzerland; 6 Computational Evolution Group, Swiss Institute of Bioinformatics, Lausanne, Switzerland; 7 Department of Biology, Howard University, Washington, DC USA; 8 Géosciences Environnement Toulouse, CNRS-Université de Toulouse III, Toulouse, France; 9 CEREGE, Aix Marseille Université, CNRS, IRD, INRA, Coll France, Aix-en-Provence, France; 10 State Key Laboratory of Geological Processes and Mineral Resources, China University of Geosciences, Wuhan, 430074 P.R. China

**Keywords:** Carbon cycle, Palaeoclimate, Natural hazards, Palaeoecology, Element cycles

Correction to: *Nature Communications* 10.1038/s41467-025-60396-y, published online 2 July 2025

In the version of the article initially published, there was an error in Fig. 1 where the range of time periods shown in the image header, now reading “251.9 ma – 246.7 ma” from left to right, were reversed in order. Additionally, in the “Reconstructing plant productivity” section, four instances of “Pt C/yr” have been amended to “Pg C/yr”. These corrections have been made to the HTML and PDF versions of the article.

