# Supplementary material for: Early Triassic super-greenhouse climate driven by vegetation collapse
Source: Nat Commun. 2025 Jul 2;16:5400. doi: 10.1038/s41467-025-60396-y (PMC12222451; doi:10.1038/s41467-025-60396-y)
Supplement: Supplementary file 1 — Supplementary Information [file 41467_2025_60396_MOESM1_ESM.pdf]

Supplementary Materials for

Early Triassic super-greenhouse climate driven by vegetation collapse

Zhen Xu *et al.*

*Corresponding author: Zhen Xu, Z.xu@leeds.ac.uk; Jianxin Yu, yujianxin@cug.edu.cn; Benjamin Mills, B.Mills@leeds.ac.uk.

The PDF file includes:

Supplementary text 1
Figures S1 to S6

Other Supplementary Materials for this manuscript include the following:

Table S1 to S7

Supplementary discussion part 1: Non-marine strata age dating and PTME correlation on land

The South China area, located in a low latitude tropical region, has many marine-terrestrial intercalations that enable high resolution chronostratigraphy for the terrestrial extinction and recovery events because marine biostratigraphic data can be tied into the successions[22]. High resolution radiometric dates are also available. Thus, a zircon radiometric age of 252.30±0.07 Ma in the terrestrial Chahe Section, together with a marine bivalve assemblage (*Pteria ussurica variabilis* - *Towapteria scythica* - *Eumorphotis venetiana)* and a spinicaudatan assemblage (*Euestheria gutta* - *Palaeoclimnadiopsis vilujensi)* helps date changes in the top of Xuanwei Formation and the bottom of the Kayitou Formation. Features in this interval, including development of the *Leiotriletes* - *Vittatina* - *Protohaploxypinus* spore assemblage, and suspected fungal spores *Veryhachium, Micrhystridium, Tympanicysta*, the disappearance of the last coal seam, a Hg/TOC spike, and wildfire proxies including a charcoal spike, all occur within the Permian-Triassic transition before the first phase of the marine extinction[11,21,45,69,71,113,122–128]. Note, in contrast, the recently published zircon data by Wu et al.[70] suggest that the land plant extinction occurred a few thousand years later than the marine extinction. Here we placed the land plant extinction in the Permian-Triassic transition, and the debate over the timing of the plant and marine extinctions does not affect the stage-level timeframe analysed in this study.

Southeastern Asia was in the low latitude southern Tethys Ocean area during the Permian-Triassic and includes North Tibet, Indonesia, the Sibumasu Plate (Tailand, Malaysia, Myanmar), Vietnam and Laos[129]. The death of the end-Changhsingian *Peltichia kwangtungensis* - *Acosarina minuta* - *Rhipidomella hessensis* - *Schuchertella* cf. *cooperi – Derbyia* - *Waagenites soochowensis* - *Spinomarginifera chenyaoyanensis* - *Marginiferinae* assemblage in the top of the Vietnam Yenduyet Formation, and the extinction of the ammonite *Pseudotirolites* and the land tetrapod *Daptocephalus* (*Dicynodon*) marked the Permian-Triassic boundary[130,131].

The Dead Sea area including Jordan, Arabia, Turkey and other areas were in low latitude western Tethyan regions during the Permo-Triassic[48,97]. The pollen *Pretricolpipollenites bharadwajii* common in the Dead Sea and higher latitude Pakistan and India indicates a possible latest Permian age for the Gondwana and Cathaysian mixed flora in these areas[48,97,132,133]. The overlying strata yield the conodont *Hadrodontina aequabilis* and foraminifera *"Cornuspira" mahajeri* suggesting an early Induan age[133]. In the southern hemisphere, middle–high latitude Kashmir area, the Permo-Triassic Boundary (PTB) is defined by the first occurrence of *Hindeodus parvus*, in the lower E2 Member of the Khunamuh Formation, together with a carbon isotope chemostratigraphy[87].

In South Africa, in the terrestrial sections of the Karoo Basin the Permian-Triassic plant collapse coincides with the extinction of *Daptocephalus* (*Dicynodon*) and the appearance of the *Lystrosaurus*, a negative carbon isotope shift, together with the disappearance of the spore *Dulhuntyispora parvithola* and the appearance of the *Playfordiaspora crenulata*[93–95,108,109,134–136]. A detrital zircon age of 252.24±0.11 Ma derived from the onset of the *Lystrosaurus* biota indicates that the plant losses were 340–370 Kyrs earlier than the marine extinction[95,137].

In the high southern latitude areas of Australia, the terrestrial crisis is marked by a plant macroflora extinction event at the base of the Zewan Group which has a zircon CA-ID-TIMS age of 252.31±0.07 Ma. This coincides with a negative carbon isotope shift, Ni/Al peaks, the

disappearance of coal, the spore *Dulhuntyispora parvithola* and the appearance of the *Playfordiaspora crenulate*. This zircon age suggests the land crisis was 410–700 Kyrs earlier than the PTB[89,138–141].

In Antarctica, the terrestrial PTB is marked by the disappearance of a Gondwana *Glossopteris* flora, appearance of the *Lystrosaurus*, and negative carbon isotope shifts[142,143]. Argentina is also found in high southern latitudes of Pangea. The PTB is placed in the top of the Puesto Tscherig Formation or the bottom of the Puesto Vera Formation with an imprecise age of 253±2 Ma in the boundary between these two formations[73,144]. The *Glossopteris* flora extinction happened around the middle Changhsingian in the South Polar area, which is much earlier than the PTB[73,145].

North China sat in mid northern latitudes of eastern Tethys. The extinction of the Voltziales flora in the region occurs within the Sunjiagou Formation, at a level dated as 252.21±0.15 Ma[84,146]. The Early Triassic spinicaudatan assemblage of *Euestheria gutta* - *Palaeolimnadiopsis vilujensis* occurs above this flora. In North China, the disappearance of coal and floral losses are 150–270 Kyrs earlier than the marine extinction, and palaeomagnetic records show that the Smithian/Spathian boundary is possibly in the upper Liujiagou Formation, whilst there is a hiatus at the Olenekian/Anisian boundary[84,146].

The PTB terrestrial records of the Xinjiang region show the replacement of late Permian tetrapods (*Striodon magnus, Jimusaria* (*Dicynodon*) *sinkianensis, Dicynodon tienshanensis)* by a *Lystrosaurus* fauna. There is also a complete turnover of the ostracod fauna; these changes predate the marine losses[91,92,147,148].

The European area we study was in the continental interior of Pangea and at a similar latitude to North China. Only the western Germanic Basin contains continuous sequences. The terrestrial PTB is placed in the bottom of the Buntsandstein Formation between the boundary of the spinicaudata *Falsisca eotriassica* assemblage and the *F. verchojanica* assemblage, the palynological *Lundbladispora obsoleta* - *Lunatisporites noviaulensis* assemblage and the *Lundbladispora willmotti* - *Lunatisporites hexagona* assemblage, which coincide with a carbon isotope negative excursion, and palaeomagnetic evidence[36,83,107,149].

In Siberia, the PTB is placed within the lower Maltsev Formation of the Kuznetsk Basin according to detrial zircon ages and magnetostratigraphy. As in Xinjiang, the ostracod fauna shows a major turnover in the lower Maltsev Formation although standing diversity remains fairly constant[49]. Angara *Cordaites* species dominate the plant assemblages below the Maltsev Formation, but they do not range up into this Formation where ferns, spenopsids and lycopsids occur instead[49].

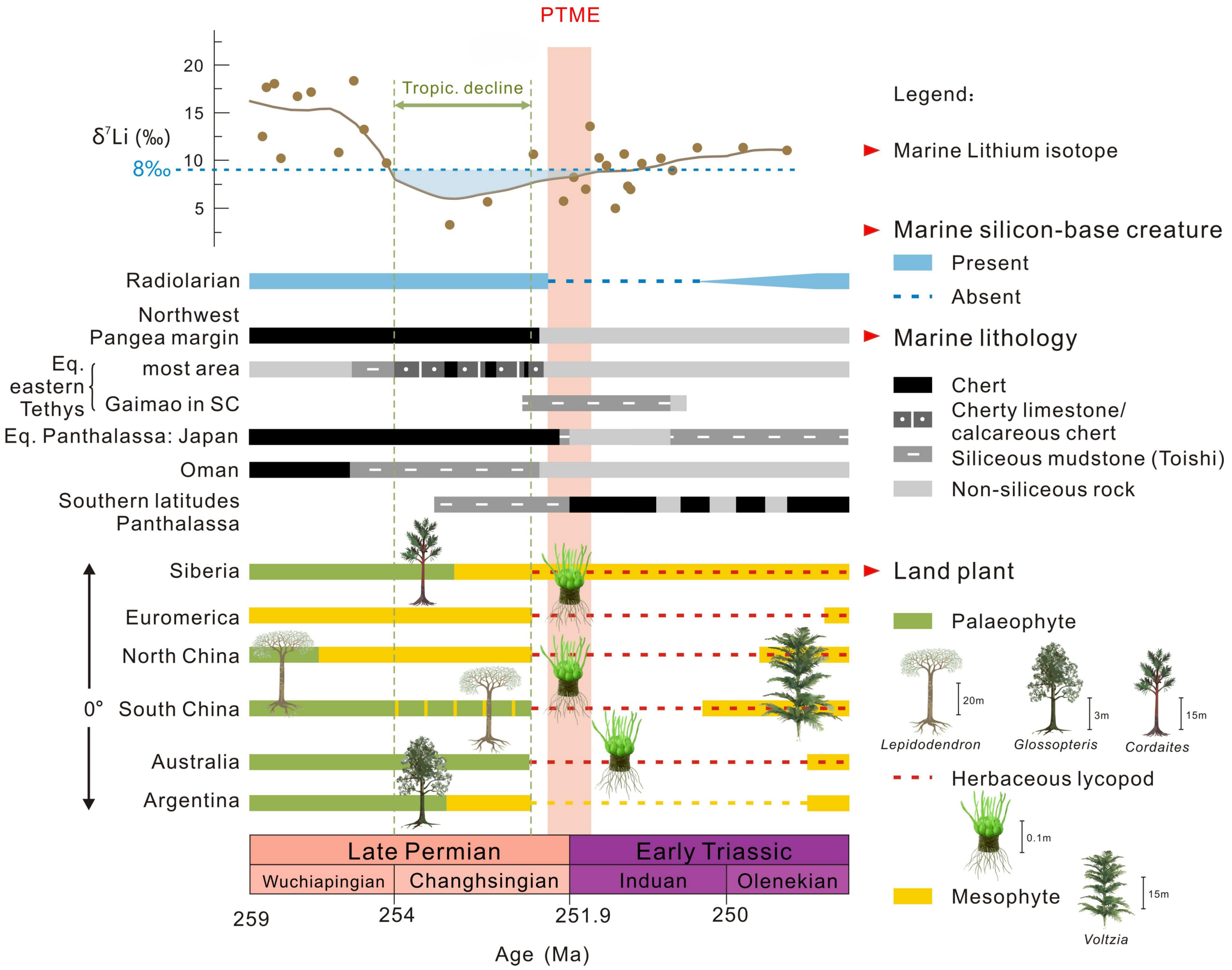

Figure S1. Summary of recent evidence showing the persistence of cherts during the PTME and the early recovery of radiolarians. The land plant extinction is the only event that continues over the whole Early Triassic hothouse. Eq.: Equatorial; SC: South China; Tropic. Decline: Tropical forest decline; PTME: Permian Triassic Mass Extinction. Lithium isotope data comes from ref.[15]. Radiolarian data comes from ref.[150]. Lithology data of global major marine sections comes from ref.[19]. All the fossil plant reconstructions are inspired after ref.[22].

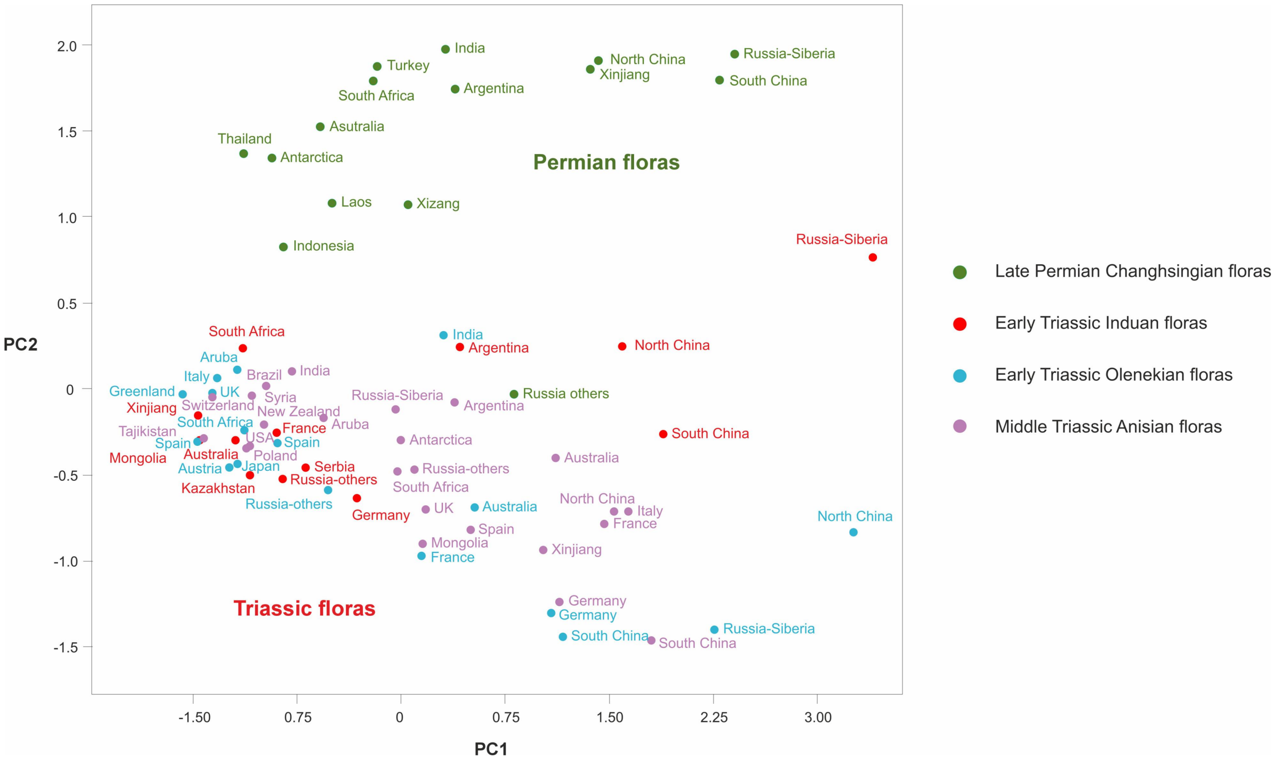

Figure S2. Two-dimensional Principal Component Analysis (PCA) of the late Permian to Middle Triassic floras in various countries. Only normalized plant macrofossils are used in this analysis.

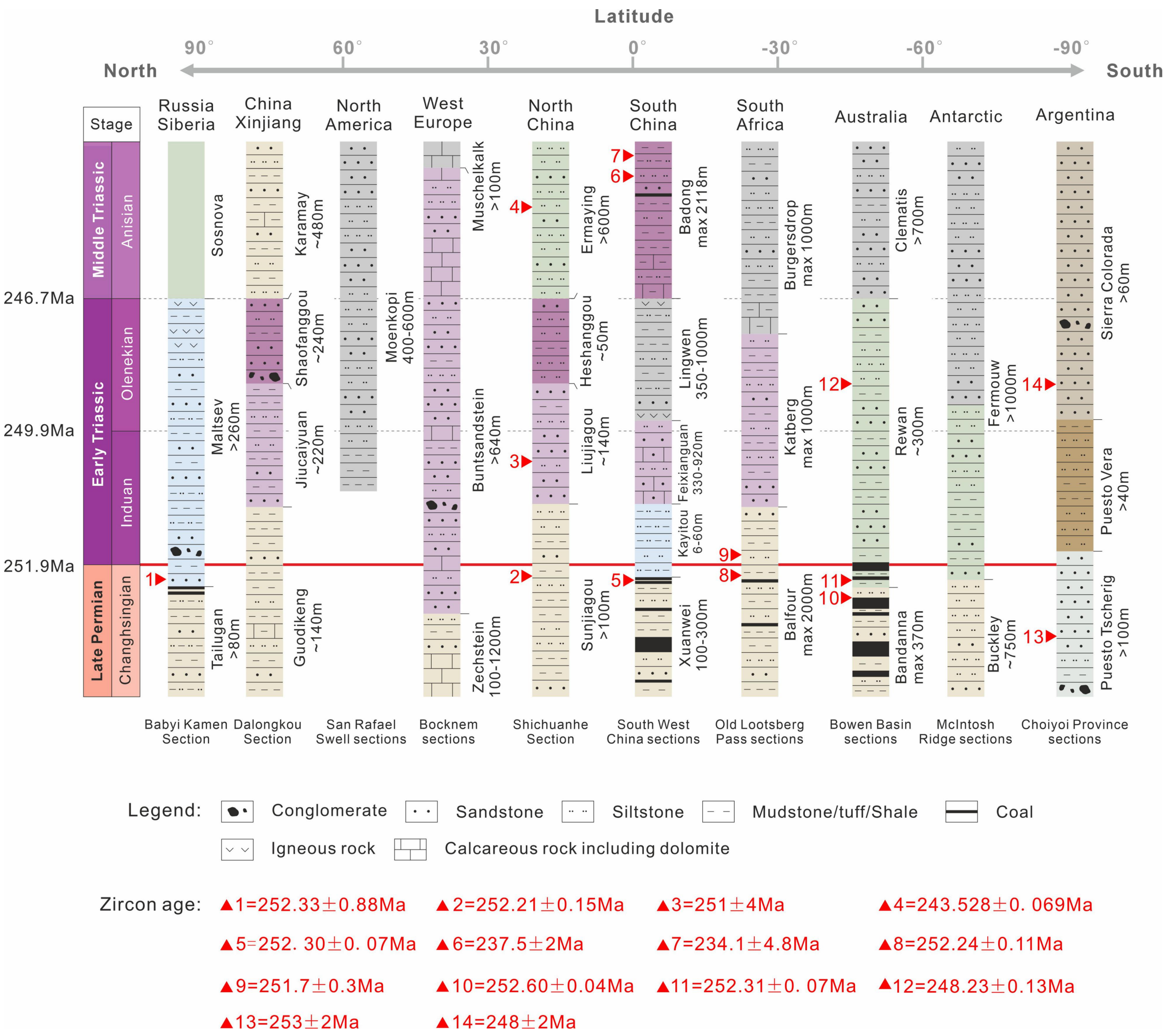

Figure S3. Global non-marine section chronostratigraphy and zircon ages. The color of each bar represents the rock color recorded in the references. SFG=Shaofanggou, HSG=Heshanggou, KYT=Kayitou, FXG=Feixianguan. The Siberian data comes from ref.[49,151]; the Xinjiang data comes from ref.[91,92,148,152,153]; the North American data comes from ref.[154]; the western European data comes from ref.[83,107,149]; the North China data comes from ref.[146,155]; the South China data comes from ref.[22,45,68,156–160]; the South African data comes from ref.[93,95,134–136,161]; the Australian data comes from ref.[89,90,138,139,141,162,163]; Antarctica data comes from ref.[142,164–168]; the Argentinian data comes from ref.[73]. See details of each basin in the supplementary text 1.

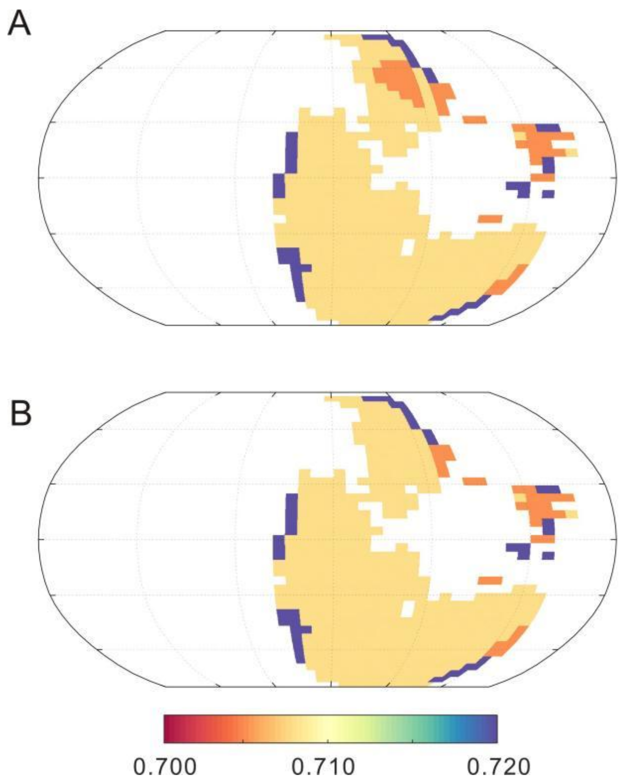

Figure S4. Model strontium isotopic $^{87}Sr/^{86}Sr$ distribution for continental gridcells. The *SCION* Earth surface is divided into 48×40 gridcells. Continental arcs have more radiogenic felsic values, whereas LIPs and suture zones have more mafic unradiogenic values. All other continental gridcells have an average sediment value. A. Early to Middle Triassic post Siberian Traps emplacement. B. Late Permian prior to Siberian Traps emplacement. See methods and text for literature sources. The palaeogeographic reconstructions are inspired by the PaleoMAP Project (http://www.scotese.com/Default.htm).

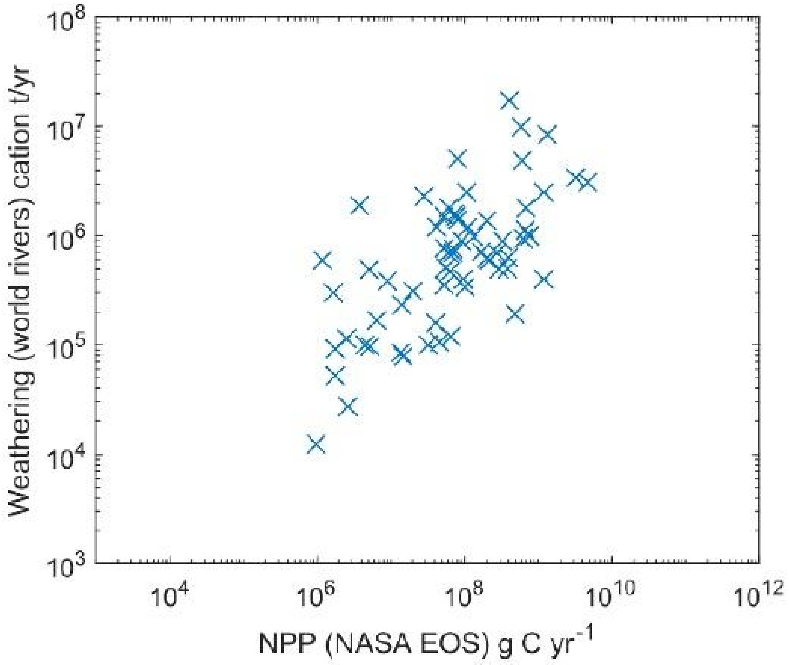

Figure S5. Recent vegetation Net Primary Productivity (NPP) and catchment scale silicate weathering relationship. From Rogger et al.[51] and Gurung et al.[52,117].

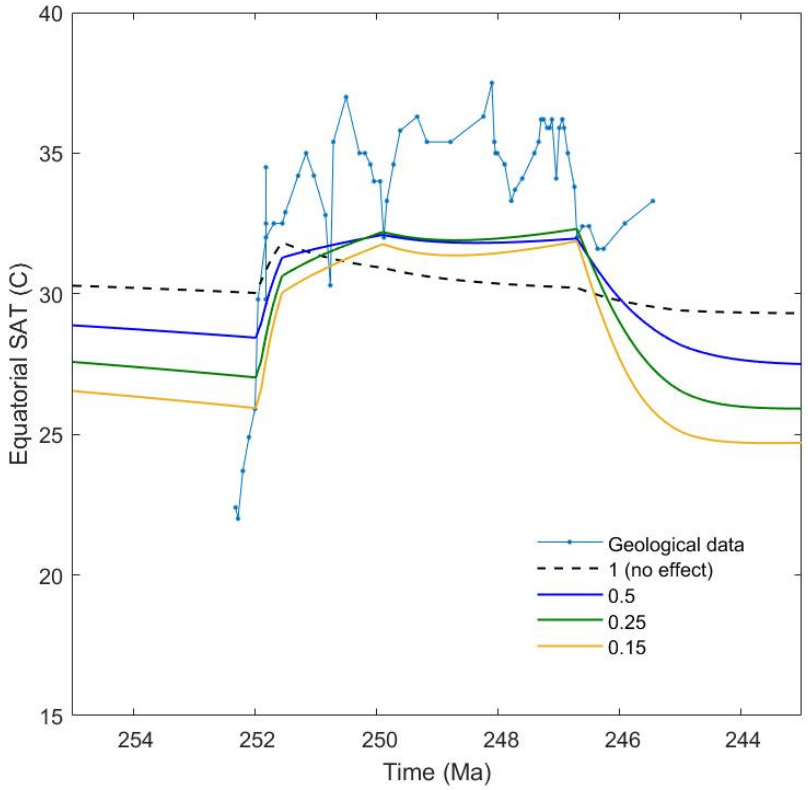

Figure S6. ***SCION*** **model outputs with different assumptions for biotic weathering enhancement.** Line colours show model runs with different values for relative 'preplant' weathering rates. The figure in the main paper uses 0.25, taken from the *GEOCARB* models. A lower preplant value results in a large temperature change when the vegetation is limited. The effect of this biotic weathering outweighs the effect of both Siberian Traps degassing and changes to organic carbon burial, which are demonstrated with the black dashed line in which biotic weathering does not change. See main text for further discussion. Blue line with dots shows temperature proxy data from ref.[10].

Table S1. Normalized plant macrofossil (bsingle) species name and major palynology (bold) genera name in location without plant macrofossil of each substage.

Changhsingian-species	Induan-species	Olenekian-species	Anisian-species
Abrotopteris guizhouensis	*Abrotopteris (Gigantonoclea) guizhouensis*	*Aethophyllum speciosum*	*Acrostichides rhombifolius*
Acrostichites concinnus	*Acitheca (=Polymorphopteris)*	*Aipteris wuziwanensis*	*Aethophyllum foetterlianum*
Acrostichites fragilis	*Acrostichites tunguskanum*	*Albertia elliptica*	*Aethophyllum speciosum*
Acrostichites kirjamkensis	*Aethophyllum* sp.	*Albertia latifolia*	*Aethophyllum stipulare*
Acrostichites linnaeaefolius	*Annalepis zeilleri*	*Albertia speciosa*	*Aipteris* sp.
Acrostichites remotus	*Annularia shirakii*	*Ammatopsis mira*	*Albertia brauni*
Acrostichites schvedovii	*Anomopteris mougeotii*	*Annularia* sp.	*Albertia elliptica*
Acrostichites shensiensis	*Anomopteris* sp.	*Anomopteris minima*	*Albertia latifolia*
Acrostichites tchunicus	*Antholithes cylindricus*	*Anomopteris mougeotii*	*Albertia ovata*
Acrostichites tunguskanus	*Araucarites* sp.	*Araucarites* sp.	*Albertia speciosa*
Alethopteris ascendens	*Arthropitys prynadae*	*Arthropitys* sp.	*Anomopteris mougeotii*
Alethopteris norinii	*Arthropitys tunguscana*	*Asterotheca szeiana*	*Anotopteris (Cladophlebis) remota*
Alethopteris sinensis	*Asterotheca radczenkoi*	*Baiera gracilis*	*Antarctipteris sclericaulis*
Amphorispermum	*Baiera* sp.	*Benxiopteris acuta*	*Anthrophyopsis* sp.
Annularia epeclissensis	*Boreopteris* sp.	*Benxiopteris densinervis*	*Araucarites agordicus*
Annularia hunanensis	*Botrychiopsis validus*	*Benxiopteris partita*	*Araucarites massalongi*
Annularia pingloensis	*Boweria* sp.	*Benxiopteris polymorpha*	*Araucarites pachyphyllus*
Annularia shirakii	*Calamites shanxiensis*	*Bernoullia zeilleri*	*Araucarites recubariensis*
Anshuncladus aduncatus	*Chiropteris* sp.	*Brachyphyllum* sp.	*Arberophyllum* sp.
Anshuncladus contiguus	*Cladophlebis borealis*	*Calamites arenaceus*	*Ashicaulis beardmorensis*
Anshuncladus xinminensis	*Cladophlebis concinna*	*Calamites shanxiensis*	*Ashicaulis woolfei*
Antholithes sp.	*Cladophlebis parvulus*	*Caulopteris parvisigillata*	*Asterotheca rigbyana*
Araucarites sp.	*Cladophlebis tajmyrensis*	*Chiropteris taizihoensis*	*Asterotheca szeiana*
Arberia sp.	*Cordaites insignis*	*Cidarophyton rewanense*	*Baiera cuyana*
Arberophyllum	*Cordaites principalis*	*Cladophlebis carnei*	*Bernoullia* sp.
Arthropitys medullatus	*Crassinervia (=Dolerophyllum) acuminata*	*Cladophlebis gaillardotii*	*Bjuvia dolomitica*
Arthropitys prynadae	*Crematopteris* sp.	*Cladophlebis gracilis*	*Brachyphyllum* sp.
Arthropitys tunguskanum	*Ctenis* sp.	*Cladophlebis ichunensis*	*Bromsgrovia willsii*
Asterophyllites sp.	*Ctenopteris angustiloba*	*Cladophlebis mendozaensis*	*Calamites aliwalensis*
Asterotheca (Pecopteris) guizhouensis	*Cylomeia* sp.	*Cladophlebis platyphylla*	*Calamites arenaceus*
Baiera sp.	*Cylostrobusclavatus*	*Cladophlebis raciborskii*	*Calamites mougeotii*
Bardella sp.	*Dicotyophyllum* sp.	*Cladophlebis roessertii*	*Calamites remotus*
Bernoullia sp.	*Dicroidium odontopteroides*	*Cladophlebis tenerus*	*Calamites shanxiensis*
Bicoemplectopteris hallei	*Discopteris* sp.	*Clathrophyllum merianii*	*Caulopteris lesangeana*
Boreopteris evenkensis	*Dzergalanella merianii*	*Coniopteris burejensis*	*Caulopteris micropeltis*
Boreopteris triangularis	*Elatides* sp.	*Coniopteris ramosa*	*Caulopteris tessellata*
Bothrodendron sp.	*Elatoclatus linearis*	*Cordaites inhofii*	*Caulopteris voltzii*
Botrychiopsis sp.	*Eleganopteris* sp.	*Cordaites mairii*	*Chiropteris barrealensis*
Boweria taimurica	*Equisetites mougeotii*	*Crematopteris typica*	*Chiropteris digitata*
Boweriar angiferina	*Equisetites sixtelae*	*Ctenozamites cycadea*	*Chiropteris zeilleri*
Calamites Schiitzeiformis	*Equisetum arenaceum*	*Ctenozamites sarranii*	*Cladophlebis densifolia*
Calamoderma sp.	*Equisetum* cf. *brongniartii*	*Cyclostrobus clavatus*	*Cladophlebis leuthardtii*
Callipteris sp.	*Equisetum mougeotii*	*Cyclostrobus sydneyensis*	*Cladophlebis linnaefolia*
Caulopteris sichuanensis	*Equisites acanthodon*	*Czekanowskia* sp.	*Cladophlebis mendozaensis*
Chansitheca kidstonii	*Euryphyllum* sp.	*Danaeopsis hughesii*	*Cladophlebis mesozoica*

Chiropteris sp.	*Fascipteris stena*	*Danaeopsis marantacea*	*Cladophlebis remota*
Cladophlebis (Pecopteris) tenuicostata	*Feildenia* sp.	*Desmiophyllum* sp.	*Cladophlebis retallackii*
Cladophlebis argutula	*Gangamopteris qinshuensis*	*Dicroidium allophyllum*	*Cladophlebis rhoifolia*
Cladophlebis augusta	*Geinitzia* sp.	*Dicroidium dubium*	*Cladophlebis sinuata*
Cladophlebis chantaica	*Germaropteris (Peltaspermum) martinsii*	*Dicroidium gopadensis*	*Compsopteris hughesii*
Cladophlebis crenulata	*Gigantonoclea guizhouensis*	*Dicroidium lancifolium*	*Coniferomyelon* sp.
Cladophlebis fuyuanensis	*Gigantopteris dentata*	*Dicroidium nidpurensis*	*Coniopteris harringtoni*
Cladophlebis grabauiana	*Gigantopteris dictyophylloides*	*Dicroidium papillosum*	*Coniopteris walkomi*
Cladophlebis haiburnensis	*Gigantopteris dictyophyllum*	*Dicroidium voiseyi*	*Cordaites inhofii*
Cladophlebis honnamakensis	*Ginkgo* sp.	*Dicroidium zuberi*	*Crematopteris typica*
Cladophlebis ichiinensis	*Ginkgoites* sp.	*Dictyophyllidites mortonii*	*Czekanowskia* sp.
Cladophlebis jeniseica	*Glossophyllum claviforme*	*Dioonitocarpidium*	*Danaeopsis fecunda*
Cladophlebis kaoiana	*Glossopteris* sp.	*Duckworthia isoeteformis*	*Darneya dentata*
Cladophlebis kirjamkensis	*Glossotheca* sp.	*Eboracia* sp.	*Darneya mougeotii*
Cladophlebis lobifera	*Glossozamites* sp.	*Edyndella* sp.	*Darneya peltata*
Cladophlebis manchurica	*Gontriglossa* sp.	*Elatocladus* sp.	*Delemaya spinulosa*
Cladophlebis nystroemii	*Heidiphyllum* sp.	*Eleganopteris* sp.	*Desmiophyllum* sp.
Cladophlebis ozakii	*Katasiopteris* sp.	*Equisetites brongniartii*	*Dicroidiopsis* sp.
Cladophlebis parapermica	*Khonomakidium tunguscanum*	*Equisetites keuperina*	*Dicroidium coriaceum*
Cladophlebis permica	*Kirjamkenia lobata*	*Equisetites mougeotii*	*Dicroidium crassinervis*
Cladophlebis permiensis	*Lepidodendron* sp.	*Equisetites mougeotii*	*Dicroidium crassum*
Cladophlebis prynadae	*Lepidopteris arctica*	*Equisetites qionghaiensis*	*Dicroidium dubium*
Cladophlebis pygmaea	*Leuthardtia crassa*	*Equisetites singularis*	*Dicroidium dutoitii*
Cladophlebis rarinervis	*Lobatannularia linearis*	*Esterella delicatula*	*Dicroidium elongatum*
Cladophlebis subfalcata	*Lobatannularia multifolia*	*Euryphyllum* sp.	*Dicroidium eskense*
Cladophlebis tenuicostata	*Lobatopteris multinervis*	*Gangamopteris qinshuiensis*	*Dicroidium fremouwensis*
Cladophlebis uralica	*Lobatopteris polymorpha*	*Gangamopteris tuncunensis*	*Dicroidium hughesii*
Cladophlebis whitbiensis	*Lutuginia* sp.	*Ginkgo marginatus*	*Dicroidium lancifolium*
Cladophlebis williamsonii	*Lycoderma* sp.	*Ginkgoites* sp.	*Dicroidium natalense*
Cladophlebis yunnanica	*Lycomeia rossica*	*Gleichenites benxiensis*	*Dicroidium odontopteroides*
Cladophlebis zwetkoviensis	*Marchajella angusta*	*Glossophyllum shensiense*	*Dicroidium pinnis-distantibus*
Comia sp.	*Marchajella kaschirzewii*	*Glossopteris browniana*	*Dicroidium prolungatum*
Compsopteris contracta	*Mertensides* sp.	*Glossopteris communis*	*Dicroidium shirleyi*
Compsopteris imparis	*Mesenteriophyllum*	*Glossopteris damudica*	*Dicroidium spinifolium*
Compsopteris multinervis	*Neokoretrophyllites linearis*	*Glossopteris gopadensis*	*Dicroidium stelznerianum*
Compsopteris wongii	*Neomariopteris* (*Sphenopteris*) *lobifolia*	*Glossopteris linearis*	*Dicroidium superbum*
Cordaites principalis	*Neuropteridium* ? *intermedium*	*Glossopteris nidpurensis*	*Dicroidium voiseyi*
Crassinervia (=*Dolerophyllum*) sp.	*Neuropteridium elegans*	*Glossopteris nilssonioides*	*Dicroidium zuberi*
Ctenis	*Neuropteridium grandifolium*	*Glossopteris papillosa*	*Dictyophyllum barrealensis*
Darneya sp.	*Neuropteridium tunguscanum*	*Glossopteris senii*	*Dictyophyllum castellanosii*
Dictyopteridium flabellatum	*Nilssonia*	*Glossopteris shanxiensis*	*Doratophyllum* sp.
Dictyopteridium sporiferum	*Noeggerathiopsis pseudominutifolia*	*Glossopteris taeniopteroides*	*Dordrechtites* sp.
Discopteris dakatensis	*Osmundopsis angusta*	*Glossotheca cochlearis*	*Equisetites arenaceus*
Discopteris rotundiloba	*Pachypteris* sp.	*Glossotheca cuneiformis*	*Equisetites conicus*
Dizeugotheca sp.	*Pagiophyllum* (*Araucarites*) *vandijkii*	*Glossotheca petiolata*	*Equisetites gracilis*
Elatides sp.	*Palaeovittaria* sp.	*Glossozamites* sp.	*Equisetites mougeotii*
Elatocladus linearis	*Paracalamites doliaris*	*Glottolepis glabrosa*	*Equisetum brongniartii*
Eleganopteris tripinnata	*Paracalamites stenocostatus*	*Glottolepis ovata*	*Fuechselia schimperi*

Equisetites sp.	*Parajacutiella* sp.	*Glottolepis rugosa*	*Ginkgo* sp.
Fascipteris hallei	*Pecopteris sulziana*	*Glottolepis sidhiensis*	*Ginkgoites dutoitii*
Fascipteris sinensis	*Pecopteris* (*Asterotheca*) *orientalis*	*Glottolepis tuberculata*	*Ginkgoites semirotunda*
Fascipteris stena	*Pelourdea* (=*Yuccites*) *jacutensis*	*Gopadia coriacea*	*Gleichenites antarcticus*
Gigantonoclea acuminatiloba	*Pelourdea* (=*Yuccites*) *vogesiacus*	*Gopadia papillata*	*Glossophyllum* sp.
Gigantonoclea dictyophylloides	*Peltaspermum calycinum*	*Helicorhiza duckworthensis*	*Gordonopteris lorigae*
Gigantonoclea guizhouensis	*Peltaspermum lobatum*	*Hymenophyllites tenellus*	*Haidingera schaurothiana*
Gigantonoclea hallei	*Peltaspermum lobutalum*	*Isoetites sagittatus*	*Hausmannia dentata*
Gigantonoclea largrelii	*Phyllotheca kryshtofovichii*	*Katasiopteris* sp.	*Hausmannia faltisiana*
Gigantonoclea lobata	*Phyllotheca yushenensis*	*Kchonomakidium* sp.	*Heidiphyllum elongatum*
Gigantonoclea longifolia	*Pityophyllum* sp.	*Kirjamkenia* sp.	*Hoegia* sp.
Gigantonoclea longmendongensis	*Pleurocaulis rewanense*	*Legnophora girardi*	*Indotheca sakesarensis*
Gigantonoclea meridionalis	*Pleuromeia altinis*	*Lepacyclotes* (=*Annalepis*) *zeilleri*	*Isoetites brandneri*
Gigantonoclea nicotianaefolia	*Pleuromeia jiaochengensis*	*Lepidopteris indica*	*Johnstonia coriacea*
Gigantonoclea plumosa	*Pleuromeia patriformis*	*Lepidopteris madagascariensis*	*Johnstonia stelzneriana*
Gigantonoclea rosulata	*Pleuromeia reniformis*	*Leuthardtia ovalis*	*Kantia* sp.
Gigantopteris cordata	*Pleuromeia sternbergii*	*Lobatannularia heianensis*	*Knorriopteris mariana*
Gigantopteris dictyophylloides	*Pleuromeia taymirica*	*Lutuginia* sp.	*Lepacyclotes* (=*Annalepis*) *angusta*
Gigantopteris dictyophyllum	*Protoblechnum* (*Compsopteris*) *contracta*	*Meristophyllum* (=*Praephylladoderma*) sp.	*Lepacyclotes* (=*Annalepis*) *bechstaedtii*
Gigantopteris meganetes	*Prynadaeopteris*	*Mertensides* sp.	*Lepacyclotes* (=*Annalepis*) *brevicystis*
Gigantopteris nicotianaefolia	*Prynadaia* sp.	*Mesenteriophyllum* sp.	*Lepacyclotes* (=*Annalepis*) *furongqiaoensis*
Gigantopteris paradoxa	*Pseudoaraucarites*	*Microphyllopteris* (=*Korallipteris*) sp.	*Lepacyclotes* (=*Annalepis*) *latiloba*
Gigantopteris ricotianaefolia	*Pseudoctenis* sp.	*Neocalamites asperrimus*	*Lepacyclotes* (=*Annalepis*) *zeilleri*
Ginkgoites sp.	*Pseudotorellia* sp.	*Neocalamites merianii*	*Lepidodendrites tessellata*
Glossophyllum sp.	*Pterophyllum* sp.	*Neocalamites shanxiensis*	*Lepidopteris africana*
Glossopteris anatolica	*Pursongia* sp.	*Neokoretrophyllites* sp.	*Lepidopteris brownii*
Glossopteris symmetrifolia	*Quadrocladus pachyphyllum*	*Neuropteridium bergense*	*Lepidopteris madagascariensis*
Glottophyllum sp.	*Quadrocladus sibiricus*	*Neuropteridium curvinerve*	*Lepidopteris murtonii*
Gontriglossa sp.	*Rhipidopsis* sp.	*Neuropteridium grandifolia*	*Lepidopteris stormbergensis*
Heidiphyllum sp.	*Schizoneura gondwanensis*	*Neuropteridium grandifolium*	*Lesangeana hasseloti*
Iniopteris sp.	*Schizoneura megaphylla*	*Neuropteridium marginatum*	*Lesangeana voltzii*
Katasiopteris lebedevii	*Schizoneura paradoxa*	*Neuropteridium voltzii*	*Lobatannularia sp.*
Katasiopteris polymorpha	*Schvedopteris lobata*	*Nidia ovalis*	*Lycopia dezanchei*
Kirjamkenia sp.	*Spehnopteris* sp.	*Nilssonia grandifolia*	*Macrotaeniopteris* sp.
Korvuntchania sp.	*Sphenobaiera porrecta*	*Noeggerathiopsis obovata*	*Marantoidea* sp.
Lebachia sp.	*Sphenobaiera tajmyrensis*	*Nymboidiantum multilobatum*	*Marattiopsis* sp.
Lelstotheca sp.	*Sphenobaiera tunguscana*	*Osmundopsis* sp.	*Microcachrydites doubingeri*
Lepidodendron (*Cathaysiodendron*) *acutangulum*	*Sphenobaiera vittaefolia*	*Otozamites vogesiacus*	*Microcachrydites sittleri*
Lepidodendron acutisquamus	*Sphenophyllum speciosum*	*Pachypteris* sp.	*Neocalamites carrerei*
Lepidodendron emeishamensis	*Sphenopteris polymorpha*	*Pagiophyllum* sp.	*Neocalamites merianii*
Lepidodendron lepidophlodes	*Sphenopteris tenuis*	*Palaeovittaria shanxiensis*	*Neocalamites shanxiensis*
Lepidodendron lepidophloides	*Sphenopteris trisecta*	*Palissya* ? sp.	*Neuropteridium bergense*
Lepidodendron lepidophylloides	*Taeniopteris ensis*	*Paracalamites* sp.	*Neuropteridium elegans*
Lepidodendron oculusfelis	*Taeniopteris prynadae*	*Pecopteris sulziana*	*Neuropteridium grandifolia*
Lepidodendron xuanweiensis	*Taeniopteris tajmyrica*	*Pecopteris whitbiensis*	*Neuropteridium grandifolium*
Lepidopteris martinsii	*Takhtajanodoxa mirabis*	*Pelourdea* (=*Yuccites*) *anastomosis*	*Neuropteridium imbricatum*
Lidgettonia africana	*Tatarina* sp.	*Pelourdea* (=*Yuccites*) *ensiformis*	*Neuropteridium intermedium*
Lidgettonia inhluzanensis	*Tersiella* sp.	*Pelourdea* (=*Yuccites*) *vogesiaca*	*Neuropteridium marginatum*

Lidgettonia lidgettonioides	*Todites korvunchanica*	*Pelourdea (=Yuccites) vogesiacus*	*Neuropteridium voltzii*
Lidgettonia mooiriverensis	*Todites orulanganensis*	*Peltaspermum calycinum*	*Neuropteris elegans*
Linophyllum xuanweiense	*Todites shensiensis*	*Peltaspermum lobulatum*	*Neuropteris grandifolia*
Linopteris brongniartii	*Tomia*	*Phyllotheca bella*	*Neuropteris imbricata*
Lixotheca permica	*Tomiostrobus (=Annalepis) augusta*	*Phyllotheca bicruris*	*Neuropteris intermedia*
Lobatannularia cathaysiana	*Tomiostrobus (=Annalepis) brevicystis*	*Phyllotheca marginans*	*Neuropteris voltzii*
Lobatannularia ensifolia	*Tomiostrobus (=Annalepis) latiloba*	*Phyllotheca yusheensis*	*Nilssonia costanervis*
Lobatannularia fusiformis	*Tomiostrobus (=Annalepis) zeilleri*	*Pinites ramosus* sp.	*Nilssonia hogardi*
Lobatannularia heianensis	*Tomiostrobus beloserovii*	*Pityophyllum* sp.	*Nilssonia reservoirensis*
Lobatannularia lingulata	*Tomiostrobus bulbosus*	*Platysaccus leschikii*	*Noeggerathiopsis* sp.
Lobatannularia multifolia	*Tomiostrobus fusiformis*	*Platysaccus queenslandi*	*Notophytum krauselii*
Lobatannularia nampoensis	*Tomiostrobus gorskyi*	*Pleurocaulis rewanense*	*Nymboidiantum glossophyllum*
Lobatannularia sichuanensis	*Tomiostrobus migayi*	*Pleuromeia epicharis*	*Oleandridium* sp.
Lobatopteris multinervis	*Tomiostrobus radiatus*	*Pleuromeia germari*	*Otozamites vogesiacus*
Lobatopteris tchalibiramica	*Tundrodendron* sp.	*Pleuromeia longicaulis*	*Pachydermophyllum dubium*
Marattiopsis? sp.	*Tungussopteris cladophleboides*	*Pleuromeia obrutschewii*	*Pachydermophyllum praecordillerae*
Mertensides bullatus	*Vetlugospermum rombicum*	*Pleuromeia oculina*	*Pachypteris* sp.
Mertensides concinnus	*Voltzia heterophylla*	*Pleuromeia olenekiensis*	*Pagiophyllum weissmanni*
Mertensides lingulatus	*Walchia* sp.	*Pleuromeia reniformis*	*Palaeoxyris regularis*
Mesenteriophyllum sp.	*Yavorskia* sp.	*Pleuromeia rossica*	*Palissya massalongi*
Neocalamites mansfeldicus	*Zuberia* sp.	*Pleuromeia sternbergii*	*Paraschizoneura jonesii*
Neokoretrophyllites annularioides	***Aculeisporites***	*Pleuromeia wuziwanensis*	*Parasciadopitys aequata*
Neokoretrophyllites linearis	***Alisporites***	*Podozamites lanceolatus*	*Parsorophyllum indicum=Dicroidium zuberi*
Neomariopteris sp.	***Apiculatisporis***	*Protoblechnum (Compsopteris) wongii*	*Pecopteris gracilis*
Neuropteridium coreanicum	***Araucariacites***	*Prynadaeopteris sp.*	*Pecopteris sulziana*
Neuropteridium guizhouensis	***Brachysaccus***	*Prynadaia sp.*	*Pelourdea (=Yuccites) anastomosis*
Neuropteridium nervosum	***Callumispora***	*Psymnophyllum multipartitum*	*Pelourdea (=Yuccites) vogesiaca*
Neuropteridium ovata	***Cedripites***	*Pterophyllum hogardii*	*Pelourdea (=Yuccites) vogesiacus*
Neuropteridium polymorphum	***Chordasporites***	*Pterozamites sinensis*	*Peltaspermum bornemannii*
Neuropteris permica	***Converrucosisporites***	*Ptilonymba*	*Peltaspermum miracarinatum*
Nillsonia sp.	***Convolutispora***	*Qionghaia carnosa*	*Peltaspermum multicostaum*
Noeggerathiopsis spathulata	***Crescentipollenites***	*Quadrocladus sp.*	*Petriellaea triangulata*
Odontopteris sp.	***Cycadopites***	*Rhabdotaenia sp.*	*Phlebopteris sp.*
Oligocarpia sp.	***Densipollenites***	*Rhacophyllum*	*Phoenicopsis elongatus*
Osmundopsis uralica	***Densoisporites***	*Rhipidopsis narrabeenensis*	*Phyllotheca sp.*
Otofolium ovatum	***Dictyophyllidites***	*Schizoneura megaphylla*	*Pinites goeppertianus*
Pachydermophyllum sp.	***Ephedripites***	*Schizoneura merianii*	*Pinites ramosus*
Pagiophyllum vandijkii	***Eretmonia***	*Schizoneura ornata*	*Pityophyllum sp.*
Palaeosmunda plenasioides	***Falcisporites***	*Schizoneura paradoxa*	*Platysaccus leschikii*
Palaeosmunda primitiva	***Foveosporites***	*Scutum* sp.	*Platysaccus papilionis*
Paracalamites australis	***Gordonispora***	*Scytophyllum bergeri*	*Platysaccus queenslandi*
Paracalamites stenocostatus	***Granulatisporites***	*Selaginellites polaris*	*Platysaccus reticulatus*
Paracalmites triassica	***Guttulapollenites***	*Sigillaria sternbergii*	*Platysaccus triassicus*
Paracalmites triassicum	***Inaperturopollenites***	*Sinozamites magnus*	*Pleuromeia germari*
Parajacutiella angusta	***Kamthisaccites***	*Sinozamites myrionervus*	*Pleuromeia hunanensis*
Parajacutiella parva	***Klausipollenites***	*Skilliostrobus australis*	*Pleuromeia marginulata*
Pecopteris affinis	***Kraeuselisporites***	*Sphenobaiera crassinervis*	*Pleuromeia oculina*
Pecopteris anderssonii	***Laevigatosporites***	*Sphenobaiera qiandianziensis*	*Pleuromeia sanxiaensis*

Pecopteris arborescens	***Lagenella***	*Sphenophyllum*	*Pleuromeia sternbergii*
Pecopteris arcuata	***Lapposisporites***	*Sphenopteris delabensis*	*Podozamites* sp.
Pecopteris calcarata	***Leiotriletes***	*Sphenopteris digitata*	*Protoblechnum* (=*Compsopteris*) sp.
Pecopteris chihliensis	***Leptolepidites***	*Sphenopteris lobifolia*	*Psaronius triasicus*
Pecopteris crenata	***Lophotriletes***	*Sphenopteris orientalis*	*Pseudoctenis barrealensis*
Pecopteris densifolia	***Lunatisporites***	*Sphenopteris yusheensis*	*Pseudoctenis brownii*
Pecopteris echinata	***Lundbladispora***	*Symopteris* (*Bernoullia*) *densinervis*	*Pseudoctenis fissa*
Pecopteris elegantula	***Lycopodiacidites***	*Symopteris* (*Bernoullia*) *zeilleri*	*Pseudoctenis groeberiana*
Pecopteris fuyuanensis	***Lycospora***	*Symopteris helvetica*	*Pseudoctenis grossa*
Pecopteris gracilenta	***Neoraistrickia***	*Taeniopteris abnormis*	*Pseudoctenis harringtoniana*
Pecopteris hemiteloides	***Osmundacidites***	*Taeniopteris ambiqua*	*Pseudoctenis propinquum*
Pecopteris lativenosa	***Pilasporites***	*Taeniopteris costiformis*	*Pseudovoltzia* sp.
Pecopteris lingulata	***Platysaccus***	*Taeniopteris glandulata*	*Psygmophyllum* sp.
Pecopteris longifoloides	***Playfordiaspora***	*Taeniopteris hainanensis*	*Pterophyllum angustum*
Pecopteris marginata	***Polycingulatisporites***	*Taeniopteris lentriculiforme*	*Pterophyllum hogardii*
Pecopteris nitida	***Proprisporites***	*Taeniopteris micronervis*	*Pterophyllum robustum*
Pecopteris norinii	***Protohaploxypinus***	*Taxites spathulatus*	*Ptilozamites sandbergeri*
Pecopteris orientalis	***Ringosporites***	*Tersiella* sp.	*Qionghaia carnosa*
Pecopteris pirae	***Simeonospora***	*Thinnfeldia feistmantelii*	*Rienitsia spathulata*
Pecopteris pseudotchichatchevii	***Staurosaccites***	*Thinnfeldia major*	*Rissikia media*
Pecopteris qingyunensis	***Striatoabieites***	*Todites shensiensis*	*Sagenopteris* sp.
Pecopteris sahnii	***Striomonosaccites***	*Tomia* sp.	*Saportaea dichotoma*
Pecopteris schoenleiniana	***Strotersporites***	*Tomiostrobus* (=*Annalepis*) sp.	*Saportaea flabellata*
Pecopteris shuanghuensis	***Uvaesporites***	*Tonchuanophyllum concinnum*	*Saportaea intermedia*
Pecopteris taiyuanensis	***Verrucosisporites***	*Tonchuanophyllum minimum*	*Schizoneura merianii*
Pecopteris tunguskana	***Verticipollenites***	*Tonchuanophyllum shensiense*	*Schizoneura paradoxa*
Pecopteris unita	***Vitreisporites***	*Tungussopteris* sp.	*Schleporia incarcerata*
Pecopteris zauronica		*Umkomasia* sp.	*Scolecopteris antarctica*
Pectiangium lanceolatum		*Vittaephyllum* sp.	*Scolopendrites grauvogelii*
Pelourdea (=*Yuccites*) *hallei*		*Voltzia acutifolia*	*Scolopendrites scolopendrioides*
Peltaspermum martinsii		*Voltzia brevifolia*	*Scytophyllum bergeri*
Permotheca sp.		*Voltzia heterophylla*	*Scytophyllum hunanense*
Petrophyllum eratum		*Voltzia heterophylla elegans*	*Scytophyllum neuburgianum*
Phylladoderma sp.		*Voltzia koenenii*	*Selaginellites leonardii*
Phyllotheca australis		*Voltzia quinquepetala*	*Sewardia* sp.
Pityospermum sp.		*Voltzia recubariensis*	*Sigillaria oculina*
Plagiozamites oblongifolius		*Voltzia walchiaeformis*	*Sigillaria sternbergii*
Pleuromeia sp.		*Voltzia weissmannii*	*Spaciinodum collinsonii*
Plumstedia gibbosa		*Voltziopsis townrowii*	*Sphallopteris* (=*Sphalmopteris*) *mougeotii*
Prionophyllopteris spiniformis		*Voltziopsis wolganensis*	*Sphenobaiera browniana*
Protoblechnum (*Compsopteris*) *contractum*		*Williamsonia lanceolobata*	*Sphenobaiera schenkii*
Protoblechnum (*Compsopteris*) *punctinervis*		*Yabeiella multinervis*	*Sphenobaiera stormbergensis*
Prynadaeopteris sp.		*Yabeilella mareyesiaca*	*Sphenobaiera tenuifolia*
Psaronius hexagonus		*Zamiopteris minima*	*Sphenobaiera ugotheriensis*
Psaronius housuoensis		*Zamites vogesiacus*	*Sphenopteris elegans*
Pseudoaraucarites sp.		***Alisporites***	*Sphenopteris myriophyllum*
Pseudoctenis sp.		***Apiculatisporis***	*Sphenopteris palmetta*
Pseudomariopteris hallei		***Baculatisporites***	*Sphenopteris schoenleiniana*

Pseudorhipidopsis sp.		***Calamospora***	*Sphenopteris voltzii*
Pseudoullmannia frumentarioides		***Cycadopites***	*Sphenozamites*
Pseudovoltzia liebeana		***Densoisporites***	*Strombergia*
Pterophyllum eratum		***Falcisporites***	*Strzeleckia gangamopteroides*
Pursongia beloussovae		***Kraeuselisporites***	*Taeniopteris ambigua*
Quadrocladus pachyphyllum		***Lycospora***	*Taeniopteris hainanensis*
Quadrocladus sibiricum		***Osmundacidites***	*Taeniopteris kelberi*
Quadrocladus sibiricus		***Rugatheca***	*Taeniopteris lentriculiformis*
Quadrocladus solmsii		***Stereisporites***	*Taxites massalongi*
Rajahia (*Danaeites*) *mirabilis*		***Striatella***	*Taxites vicentinus*
Rajahia (*Danaeites*) *rigida*		***Uvaesporites***	*Taxodites* (=*Glyptostrobus*) *saxolympiae*
Rajahia calceiformis		***Verrucosisporites***	*Telemachus elongatus*
Rajahia guizhouensis		***Vitreisporites***	*Tersiella* sp.
Rajahia major		***Williamsoniella***	*Thamnopteris vogesiaca*
Rajahia mirabilis			*Thinnfeldia nordenskioldii*
Rajahia rigida			*Todites pattinsoniorum*
Raniganjia kilburnensis			*Todites shensiensis*
Raniganjia sp.			*Tomaniopteris katonii*
Rhaphidopteris sp.			*Tongchuanophyllum* sp.
Rhipidopsis ginkgoides			*Townrovia petasata*
Rhipidopsis lobata			*Townroviamites brookvalensis*
Rhipidopsis lobulata			*Ullmannia brandtii*
Rhipidopsis multifurcata			*Umkomasia distans*
Rhipidopsis panii			*Umkomasia polycarpa*
Saportaea sp.			*Umkomasia resinosa*
Schizoneura brevifolia			*Umkomasia sessilis*
Schizoneura keboense			*Voltzia acutifolia*
Schizoneura manchuriensis			*Voltzia brevifolia*
Schizoneura sino-coreanum			*Voltzia curtifolia*
Schvedopteris lobata			*Voltzia elegans*
Scolecopteris guizhouensis			*Voltzia heterophylla*
Scopus confertus			*Voltzia krappitzensis*
Scopus didiscus			*Voltzia recubariensis*
Scopus gibbosus			*Voltzia walchiaeformis*
Scopus obscurus			*Williamsonia* sp.
Scutum sp.			*Xylopteris elongata*
Scytophyllum tenuinerve			*Yabeiella* sp.
Selaginellites tibeticus			*Yelchophyllum omegapetiolaris*
Shuichengella (*Cryptonoclea*) *primitiva*			*Zamites vogesiacus*
Sigillaria guizhouensis			*Zuberia barrealensis*
Spehnopteris sp.			*Zuberia feistmanteli*
Spenophyllum thonii			*Zuberia sahnii*
Sphenarion sp.			*Zuberia zuberi*
Sphenobaiera sp.			***Aculeisporites***
Sphenophyllum koboense			***Aequitriradites***
Sphenophyllum sino-coreanum			***Alisporites***
Sphenophyllum speciosum			***Apiculatisporis***
Sphenopteris lobifolia			***Cycadopites***

Sphenopteris matgitecta			***Deltoidospora***
Sphenopteris mircophylla			***Densoisporites***
Sphenopteris rotunda			***Enzonalasporites***
Sphenopteris simplicinervis			***Falcisporites***
Sphenopteris tembentchiensis			***Klausipollenites***
Sphenopteris tenuis			***Kraeuselisporites***
Sphenopteris trisecta			***Lycospora***
Stiphorus sp.			***Ovalipollis***
Szea (*Cladophlebis*) *sinensis*			***Pinuspollenites***
Szecladia multinervia			***Pityosporites***
Taeniopteris crassinervis			***Reticulatisporites***
Taeniopteris densisstma			***Sulcatisporites***
Taeniopteris dongluoensis			***Verrucosisporites***
Taeniopteris fusuiensis			***Vitreisporites***
Taeniopteris guangxiensis			
Taeniopteris multinervis			
Taeniopteris nystroemii			
Taeniopteris rarinervis			
Taeniopteris sichuanensis			
Taeniopteris szei			
Taeniopteris tajluganensis			
Takhtajanodoxa mirabilis			
Tatarina sp.			
Thinnfeldia sp.			
Tingia guadii			
Tingia hamaguchi			
Todites augusta			
Todites borealis			
Todites crenata			
Todites ichiinensis			
Todites korvunchanica			
Todites lobifera			
Todites polkini			
Todites wongii			
Tomia malzevskiana			
Tomia radczenkovii			
Tungussopteris sphenopteroides			
Ullmannia bronnii			
Ullmannia frumentaria			
Voltzia avamica			
Voltzia chachlovii			
Walchia sp.			
Wumengopteris crassirachis			
Yavorskyia arctica			
Yavorskyia radczenkovii			
Yavorskyia serrata			
Yuania magnifolia			
Zamiopteris glossopteroides			

Zhutheca (*Fascipteris*) *densata*			
Zuberia sp.			
Alisporites			
Arberiella			
Corisaccites			
Crescentipollenites			
Cycadopites			
Ephedripites			
Eretmonia			
Falcisporites			
Faunipollenites			
Guttulapollenites			
Klausipollenites			
Lunatisporites			
Pityosporites			
Platysaccus			
Striatopodocarpites			
Strotersporites			
Weylandites			

Table S2. Plant fossil location of each substage.

End Permian Changhsingian			Early Triassic Induan			Early Triassic Olenekian			Middle Triassic Anisian		
Country	Longitude	Latitude	Country	Longitude	Latitude	Country	Longitude	Latitude	Country	Longitude	Latitude
Antarctica	67.07	-72	Australia	148	-24.83	America	-110.8	35	Antarctica	159.2	-78.1
Argentina	-68.1	-28.8	Australia	148.9	-23.64	Aruba	159.7	-75.7	Antarctica	159.7	-76.7
Argentina	-66.4	-38.1	North China	109.19	35.39	Australia	114.6	-28.8	Antarctica	160.5	-77.3
Argentina	-67.4	-40.7	CN-Xinjiang	88.8	43.95	Australia	115.9	-32	Antarctica	164	-84.4
Argentina	-66.7	-48.2	North China	111.2	36.4	Australia	145.5	-17.4	Antarctica	166.4	-84.3
Austria	13.65	47.53	North China	111.86	37.24	Australia	146.9	-31.3	Antarctica	171	-83.8
Australia	147.3	-24.7	North China	111.89	37.29	Australia	149.2	-23.5	Argentina	-69.5	-31.6
Australia	148	-25	North China	112.16	37.56	Australia	151	-34.2	Argentina	-67.8	-30.2
Australia	148.8	-23.3	North China	112.2	32.5	Australia	151.3	-33.6	Argentina	-69.5	-35.6
Australia	142.7	-20.92	North China	112.3	37.3	Australia	152.8	-31.6	Argentina	-69.2	-33
South China	101.34	24.48	North China	112.85	35.49	Austria	12.8	46.8	Aruba	173	-80
South China	102.81	30.26	North China	112.9	37.1	Austria	10.7	46.6	Australia	146.9	-31.3
South China	103.04	28.51	North China	120.8	41	France	0.2	45	Australia	148.7	-32.4
South China	104	26.82	North China	122.53	41.94	France	3.3	43.7	Australia	150.3	-33.4
South China	104.26	25.68	North China	125.8	44.1	France	6.5	48.6	Australia	151.3	-33.8
South China	104.47	25.71	South China	101.34	24.48	France	6.6	48	Australia	146.8	-42.5
South China	104.51	28.16	South China	103.04	28.51	France	7.4	48.6	Australia	148.6	-32.3
South China	104.69	25.8	South China	104	26.82	France	7.5	48.4	Australia	151.2	-33.9
South China	104.8	26.7	South China	104.09	26.21	France	7.8	49	Australia	152.4	-27.1
South China	104.93	32.24	South China	104.44	26.73	Germany	6.3	50.6	Australia	152.7	-29.9
South China	104.96	26.54	South China	104.47	25.71	Germany	6.4	48.2	Brazil	-53.7	-29.6
South China	105.1	26.03	South China	104.69	25.8	Germany	6.5	50.3	Canada	-117.3	56.2
South China	105.23	25.87	South China	105.34	27.62	Germany	6.6	49.8	Egypt	34.4	30.4
South China	105.34	27.62	South China	105.38	27.17	Germany	6.7	49.4	France	4.7	46.3
South China	105.38	27.17	South China	105.46	32.31	Germany	7	49.2	France	6	47
South China	105.46	32.31	South China	107.29	26.84	Germany	7.1	49.2	France	6	48.1
South China	105.84	26.42	South China	107.31	26.93	Germany	7.3	48.9	France	6.3	48.1
South China	105.9	26.3	South China	112.29	30.27	Germany	7.4	49.2	France	6.5	48.6
South China	105.9	32.4	South China	117.92	26.48	Germany	7.7	52.3	France	6.6	48
South China	105.93	26.33	Germany	9.4	51.5	Germany	8.2	48.3	France	6.7	48.2
South China	105.95	26.25	Germany	9.4	51.6	Germany	8.4	48.9	France	6.9	49.1
South China	106.08	25.83	Germany	9.4	51.8	Germany	8.5	48.5	France	7.2	48.7
South China	106.4	29.5	Germany	9.6	51.7	Germany	8.7	49.3	France	7.2	47.9
South China	106.4	29.5	Germany	9.6	51.9	Germany	9.2	48.8	France	7.3	48.8
South China	106.43	29.92	Germany	10	51.5	Germany	9.4	51.7	France	7.4	48.7
South China	106.6	26.2	Germany	10.1	50.2	Germany	9.5	52	France	7.5	48.4
South China	107.31	26.93	Germany	11.1	50.7	Germany	9.7	50	Germany	2.8	42.6
South China	107.9	22.64	Germany	11.2	50.8	Germany	9.9	51.5	Germany	6.5	50.7
South China	108.45	31.75	Germany	11.8	51.5	Germany	10	51.4	Germany	6.6	50.6
South China	108.64	24.49	Germany	11.8	51.8	Germany	10.1	52.1	Germany	6.6	49.4
South China	108.81	23.72	Spain	-1.2	41.5	Germany	10.4	49.5	Germany	6.7	50.3
South China	108.86	23.77	Greenland	-23	73	Germany	11.1	50.7	Germany	6.7	49.4
South China	108.96	23.61	Hungary	17.61	46.88	Germany	11.1	50.3	Germany	6.8	49.3
South China	109.07	30.45	Ireland	-6.79	53.9	Germany	11.4	50.7	Germany	7	49.2
South China	109.32	23.7	Italy	11.63	46.57	Germany	11.5	51.6	Germany	7.3	48.8

South China	109.4	24.3	Norway	29.28	71.16	Germany	11.6	50.7	Germany	7.6	47.6
South China	110.88	29.42	Norway	28.84	71.24	Germany	11.7	51.8	Germany	7.8	49
South China	112.29	30.27	Norway	26.84	71.24	Germany	11.7	52	Germany	7.9	49.5
South China	112.3	22.2	Norway	22	78	Germany	11.8	51.8	Germany	8	50.3
South China	112.4	25	Poland	20.67	50.83	Germany	11.8	51.6	Germany	8.2	48.3
South China	113.76	23.38	Serbia	23	43.5	Germany	12.2	46.7	Germany	8.4	50
South China	116.1	24.3	RU-other	36.37	56.73	Greenland	-21	73.5	Germany	8.5	48.1
South China	119.93	30.9	RU-other	40.5	44	Greenland	-23	72.5	Germany	8.5	49
South China	119.99	30.92	RU-other	45	45	Greenland	-23.6	72.6	Germany	8.6	49
North China	98.4	39.5	RU-other	46.84	48.14	Greenland	-42.6	71.7	Germany	8.7	48.7
North China	99.6	38.8	RU-other	46.58	53.12	Hungary	18.1	46.1	Germany	8.8	49.4
North China	100.92	40.71	RU-other	45.91	57.88	Hungary	17.6	46.9	Germany	9.2	48.8
North China	102.2	38.5	RU-other	60	67	Hungary	17.7	47.3	Germany	9.4	51.7
North China	106.91	33.08	Russia-Siberia	82.22	72.97	India	81.9	23.8	Germany	9.5	52
North China	111.1	39	Russia-Siberia	86.5	54.5	India	81.2	23	Germany	9.6	51.7
North China	111.89	37.29	Russia-Siberia	99.29	61.06	India	77.8	23.2	Germany	9.7	50
North China	112.2	34.5	Russia-Siberia	92.18	62.64	India	81.8	24.2	Germany	9.9	51.5
North China	113.39	34.3	Russia-Siberia	152.41	62.94	Israel	34.8	31.7	Germany	10.1	50.1
North China	119.6	39.9	Russia-Siberia	98.27	63.68	Israel	34.7	31.6	Germany	10.2	48.3
North China	120.8	40.7	Russia-Siberia	107.78	63.93	Israel	34.6	31.7	Germany	10.3	49.6
North China	122.53	41.94	Russia-Siberia	98	64	Italy	12	46.5	Germany	11.1	50.7
North China	125.9	41.7	Russia-Siberia	90.84	64.71	Japan	141.8	38.8	Germany	11.4	50.9
North China	128.9	47.7	Russia-Siberia	109	65.5	Kazakhstan	118.9	63.4	Germany	11.5	51.6
CN-Xinjiang	81.8	41.8	Russia-Siberia	101.5	66	Madagascar	48.2	-14.6	Germany	11.7	52
CN-Xinjiang	85.7	43.9	Russia-Siberia	110.34	66.55	North China	96.5	42	Germany	12.2	46.7
CN-Xinjiang	89.4	42.3	Russia-Siberia	125.84	67.16	North China	99.6	38.8	Germany	12.4	48.8
CN-Xinjiang	89	43.3	Russia-Siberia	128.14	67.58	North China	102.7	25	India	72.4	32.6
CN-Xinjiang	90.4	44.7	Russia-Siberia	90.37	67.78	North China	111.2	38.5	India	77.8	23.2
CN-Xinjiang	88.8	43.95	Russia-Siberia	127.43	67.96	North China	111.9	37.3	India	81.3	24.5
CN-Xizang	86.8	33.6	Russia-Siberia	90	68	North China	112	36.4	India	87.4	22.4
Germany	9.11	50.29	Russia-Siberia	127	68	North China	112.2	37.2	Israel	34.6	31.7
Germany	9.12	50.3	Russia-Siberia	126.5	69.1	North China	112.3	35.2	Italy	10.5	45.8
Germany	9.12	50.32	Russia-Siberia	128.52	71	North China	112.9	35.5	Italy	11	46.5
Germany	9.15	50.37	Russia-Siberia	94.96	74.19	North China	112.9	37.1	Italy	11.2	45.7
Germany	10	51.15	Russia-Siberia	97.86	74.35	North China	116.1	36.1	Italy	11.2	46.4
Germany	10.02	51.67	Russia-Siberia	107.76	74.85	North China	117.9	41	Italy	11.4	45.9
Germany	10.22	51.95	Russia-Siberia	112.57	75.29	North China	122.5	41.9	Italy	11.6	46.4
Germany	10.67	52.12	Russia-Siberia	99.33	75.69	North China	123.7	41.5	Italy	12	46.3
Greenland	24.5	38.85	Russia-Siberia	126.63	75.96	Norway	23	78	Italy	12.1	46.7
Hungary	18.62	47.42	Russia-Siberia	98	79.52	Russia-other	46.8	48.2	Italy	13.3	46.5
India	87.1	24	Kazakstan	118.94	63.36	Russia-other	61.9	56.7	Mongolia	102.6	47.9
Indonesia	136.8	-4.2	Mongolia	104.29	43.5	Russia-Siberia	83.1	73.2	Mongolia	104	48.1
Italy	11.63	46.57	South Africa	29.17	-28.5	Russia-Siberia	89.7	67.1	New Zealand	169.8	-46.4
Italy	11.65	46.37	South Africa	29.33	-28.67	Russia-Siberia	90	68	New Zealand	170.2	-44.7
Italy	11.73	46.56	South Africa	29.67	-28.83	Russia-Siberia	93	65.6	North China	109.2	35.4
Japan	141.4	38.79	South Africa	29.83	-29	Russia-Siberia	93.5	68	North China	122.5	41.9
Laos	102	20	South Africa	30	-29.17	Russia-Siberia	93.9	73.5	North China	110.7	37
Pakistan	72.2	32.48	South Africa	30	-29.5	Russia-Siberia	95.6	64.1	North China	111	39.7
RU-other	40.7	43.96	South Africa	30.17	-29.17	Russia-Siberia	98	64	North China	113.6	37.4
RU-other	42.1	56.24	Russia-Siberia	87.41	54.76	Russia-Siberia	99.3	61.1	North China	123.7	41.3

RU-other	42.11	56.26	Argentina	-69	-29.7	Russia-Siberia	101.5	66	Poland	20.1	50.2
RU-other	44.8	58.9	Argentina	-70.8	-31.5	Russia-Siberia	103.5	62.2	Poland	18	50.5
RU-other	44.8	59.8	Argentina	-69.7	-33.2	Russia-Siberia	112.7	74.9	Poland	20.9	51
RU-other	46.58	53.12	Argentina	-69.2	-35.7	Russia-Siberia	115.9	60.9	Russia-others	55.1	51.8
RU-other	46.7	58.5	Argentina	-67.4	-40.7	Russia-Siberia	131.9	42.9	Russia-others	44.9	44.7
RU-other	46.9	60.8	France	4	44	Russia-Siberia	132	43.5	Russia-others	58.6	65.8
RU-other	46.9	60.8				Russia-Siberia	144.9	61.9	Russia-Siberia	126.5	69.1
RU-other	48.3	61.2				South Africa	22.5	-32.4	South Africa	22.5	-32.4
RU-other	48.9	58.6				South China	110.4	19.3	South Africa	27.1	-31.7
RU-other	52.48	54.12				South China	110	19.6	South Africa	26	-31
RU-other	57.4	65				South China	110.5	19.2	South Africa	26.5	-30.8
Russia-Siberia	90.84	64.71				Spain	-0.4	39.9	South Africa	26.7	-30.7
Russia-Siberia	99.29	61.06				Spain	2.3	41.8	South Africa	27	-31
Russia-Siberia	99.33	75.69				Tunisia	11	33	South Africa	27.3	-31.7
Slovenia	13.92	46.02				UK	-3	53.4	South China	102.8	30.3
Thailand	101	16				Russia-Siberia	87.4	54.8	South China	108.9	30.3
Turkey	40.78	38.25							South China	109.1	29.7
UK	-2.48	54.57							South China	109.5	31
UK	-2.48	54.58							South China	110	19.6
UK	-1.27	52.98							South China	110.2	29.5
USA	-104.23	31.6							South China	110.3	31
USA	-104.23	31.61							South China	111.9	27.6
USA	-104.23	31.62							South China	112.2	30.7
USA	-104.22	31.63							South China	113.9	29.7
USA	-104.21	31.65							South China	116.8	30.7
USA	-104.19	31.66							South China	119.8	33.1
USA	-104.18	31.65							South China	106.7	26.3
South Africa	22.54	-32.36							South China	106.9	23.8
South Africa	24.87	-31.92							South China	108.7	28.8
South Africa	26.03	-32.08							South China	112.6	24.7
South Africa	27.84	-26.41							Spain	-5.4	43.3
South Africa	29.46	-25.77							Spain	-4.5	36.8
South Africa	29.67	-28.83							Spain	-4.4	41.8
South Africa	29.78	-29.83							Spain	-1.8	41.7
South Africa	29.79	-29.8							Spain	-1.5	43.2
South Africa	29.83	-29							Spain	-1	40.7
South Africa	29.86	-29.01							Spain	-0.9	41.6
South Africa	30	-29.33							Spain	-0.6	40
South Africa	30	-29.17							Spain	-0.5	39.8
South Africa	30.17	-29.5							Spain	1	42.7
South Africa	32	-28.5							Spain	1.8	41.6
									Spain	2.6	42.2
									Spain	3	39.7
									Spain	-1.6	41.7
									Switzerland	7.6	47.6
									Syria	41.6	37.1
									Tajikstan	70.4	38.3
									Turkey	26.6	38.4
									Turkey	33.7	41.9
									UK	-2.1	52.3

									UK	-1.7	52.3
									UK	-1.6	54.8
									UK	-2.1	52.3
									USA	-71.4	42.4
									USA	-110.3	34.9
									USA	-105.1	35.2
									CN-Xinjiang	88.8	44
									CN-Xinjiang	81.8	41.8
									CN-Xinjiang	84.8	45.7
									CN-Xinjiang	88.2	44.2

Table S3. Land tetrapod fossil occurrence of each substage.

Substage	Country-area	Longitude	Latitude	Taxa
Changhsingian	China-Xinjiang	87.83	43.82	Diictodon feliceps
Changhsingian	China-Xinjiang	87.84	43.83	Jimusaria sinkianensis
Changhsingian	China-Xinjiang	88.83	44	Dalongkoua fuae
Changhsingian	China-Xinjiang	88.88	43.32	Turfanodon bogdaensis
Changhsingian	China-Xinjiang	88.88	44.08	Turfanodon bogdaensis
Changhsingian	China-Xinjiang	89.17	42.92	Jimusaria sinkianensis
Changhsingian	China-Xinjiang	89.17	42.92	Lystrosaurus robustus
Changhsingian	Niger	7.2	18.78	Bunostegos akokanensis
Changhsingian	Niger	7.22	18.79	Dromopus
Changhsingian	Niger	7.22	18.79	Hyloidichnus
Changhsingian	Niger	7.2	18.78	Moradisaurus grandis
Changhsingian	Niger	7.53	18.51	Moradisaurus grandis
Changhsingian	Niger	7.22	18.79	Pachypes
Changhsingian	Niger	7.2	18.78	Rubidginae
Changhsingian	Niger	7.53	18.51	Rubidginae
Changhsingian	North China	110.82	37.41	Pareiasauria
Changhsingian	North China	111.11	39.03	Pareiasauria
Changhsingian	North China	110.82	37.41	Pareiasauridae
Changhsingian	North China	110.82	37.41	Sanchuansaurus pygmaeus
Changhsingian	North China	110.82	37.41	Shihtienfenia permica
Changhsingian	North China	110.88	37.43	Shihtienfenia permica
Changhsingian	North China	111.11	39.03	Shihtienfenia permica
Changhsingian	North China	110.82	37.41	Therapsida
Changhsingian	Russia	54.91	51.3	Brontopus giganteus
Changhsingian	South Africa	23.89	-31.91	Cyonosaurus kitchingi
Changhsingian	South Africa	23.89	-31.91	Daptocephalus leoniceps
Changhsingian	South Africa	25.97	-30.5	Lystrosaurus maccaigi
Changhsingian	South Africa	26.27	-30.42	Lystrosaurus maccaigi
Changhsingian	South Africa	23.89	-31.91	Milleretta rubidgei
Changhsingian	South Africa	23.89	-31.91	Oudenodon bainii
Changhsingian	South Africa	23.89	-31.91	Procynosuchus delaharpeae
Changhsingian	South Africa	23.89	-31.91	Theriognathus microps
Changhsingian	Tanzania	39.01	-5.05	Tangasaurus mennelli
Changhsingian	UK	-3.37	57.66	Gordonia traquairi
Changhsingian	UK	-3.37	57.66	Elginia mirabilis
Changhsingian	UK	-3.37	57.66	Geikia elginensis
Induan	Australia	148	-24.83	Dicynodontia
Induan	Australia	150.91	-34.36	Dicynodontipus bellambiensis
Induan	Australia	148	-24.83	Eomurruna yurrgensis
Induan	Australia	148.9	-23.64	Eomurruna yurrgensis
Induan	Australia	148	-24.83	Kadimakara australiensis
Induan	Australia	148	-24.83	Kalisuchus rewanensis
Induan	Australia	148	-24.83	Kudnu mackinlayi
Induan	Australia	147.3	-42.88	Tasmaniosaurus triassicus
Induan	Australia	148.9	-23.64	Tasmaniosaurus triassicus

Induan	China-Xinjiang	88.83	44	Chasmatosaurus yuani
Induan	China-Xinjiang	88.88	44.08	Chasmatosaurus yuani
Induan	China-Xinjiang	88.83	44	Lystrosaurus broomi
Induan	China-Xinjiang	88.83	44	Lystrosaurus hedini
Induan	China-Xinjiang	89.17	42.92	Lystrosaurus hedini
Induan	China-Xinjiang	89.7	43.98	Lystrosaurus shichanggouensis
Induan	China-Xinjiang	88.83	44	Lystrosaurus youngi
Induan	China-Xinjiang	89.7	43.98	Lystrosaurus youngi
Induan	China-Xinjiang	88.88	44.08	Prolacertoides jimusarensis
Induan	China-Xinjiang	88.88	44.08	Santaisaurus yuani
Induan	China-Xinjiang	88.83	44	Sungeodon kimkraemerae
Induan	China-Xinjiang	87.57	43.8	Urumchia lii
Induan	Poland	16.41	50.42	Microcnemus
Induan	Russia	45.08	57.42	Blomosaurus ivachnenkoi
Induan	Russia	52.69	52.37	Chasmatosuchus
Induan	Russia	55.72	51.34	Chasmatosuchus
Induan	Russia	45.91	57.88	Crocopoda
Induan	Russia	45.26	56.94	Lystrosaurus georgi
Induan	Russia	45.08	57.42	Microcnemus
Induan	Russia	45.91	57.88	Microcnemus
Induan	Russia	55.72	51.34	Microcnemus
Induan	Russia	50.43	52.85	Phaanthosaurus
Induan	Russia	45.91	57.88	Phaanthosaurus ignatjevi
Induan	Russia	45.15	57.13	Phaanthosaurus simus
Induan	Russia	45.91	57.88	Phaanthosaurus simus
Induan	Russia	47.96	59.14	Phaanthosaurus simus
Induan	Russia	52.44	52.79	Procolophonidae
Induan	Russia	52.96	52.32	Reptilia
Induan	Russia	55.91	52.16	Reptilia
Induan	Russia	57.54	65.99	Scalopognathus multituberculatus
Induan	Russia	45.91	57.88	Vonhuenia friedrichi
Induan	South Africa	26.27	-30.42	Dicynodontipus
Induan	South Africa	26.27	-30.42	Dolomitipes
Induan	South Africa	26.27	-30.42	Dolomitipes accordii
Induan	South Africa	24.55	-31.87	Galesaurus planiceps
Induan	South Africa	26.06	-30.47	Galesaurus planiceps
Induan	South Africa	24.87	-31.37	Lystrosaurus
Induan	South Africa	26.27	-30.42	Lystrosaurus
Induan	South Africa	24.55	-31.87	Lystrosaurus curvatus
Induan	South Africa	25.7	-32.12	Lystrosaurus curvatus
Induan	South Africa	25.97	-30.5	Lystrosaurus curvatus
Induan	South Africa	26.06	-30.47	Lystrosaurus curvatus
Induan	South Africa	24.55	-31.87	Lystrosaurus declivis
Induan	South Africa	24.86	-31.84	Lystrosaurus declivis
Induan	South Africa	24.95	-31.18	Lystrosaurus declivis
Induan	South Africa	25.7	-32.12	Lystrosaurus declivis
Induan	South Africa	25.97	-30.5	Lystrosaurus declivis
Induan	South Africa	26.06	-30.47	Lystrosaurus declivis

Induan	South Africa	26.27	-30.42	Lystrosaurus declivis
Induan	South Africa	25.7	-32.12	Lystrosaurus maccaigi
Induan	South Africa	24.55	-31.87	Lystrosaurus murrayi
Induan	South Africa	24.95	-31.18	Lystrosaurus murrayi
Induan	South Africa	25.07	-30.7	Lystrosaurus murrayi
Induan	South Africa	25.7	-32.12	Lystrosaurus murrayi
Induan	South Africa	25.97	-30.5	Lystrosaurus murrayi
Induan	South Africa	26.06	-30.47	Lystrosaurus murrayi
Induan	South Africa	26.27	-30.42	Lystrosaurus murrayi
Induan	South Africa	24.86	-31.84	Moschorhinus kitchingi
Induan	South Africa	26.27	-30.42	Moschorhinus kitchingi
Induan	South Africa	25.38	-31.76	Noteosuchus colletti
Induan	South Africa	24.95	-31.18	Phonodus dutoitorum
Induan	South Africa	24.86	-31.84	Progalesaurus lootbergensis
Induan	South Africa	29.13	-28.28	Prolacerta broomi
Induan	South Africa	25.04	-30.92	Rhynchosauroides
Induan	South Africa	26.27	-30.42	Rhynchosauroides
Induan	South Africa	25	-31.73	Saurodektes rogersorum
Induan	South Africa	26.06	-30.47	Saurodektes rogersorum
Induan	South Africa	24.95	-31.18	Tetracynodon darti
Induan	South Africa	29.13	-28.28	Tetracynodon darti
Induan	South China	107.58	28.83	Chirotherium
Olenekian	Argentina	-68.05	-29.57	Chirotheriidae
Olenekian	Canada	-121.67	54.53	Wapitisaurus problematicus
Olenekian	Germany	9.17	51.33	Protochirotherium wolfhagense
Olenekian	Germany	10.01	51.47	Ctenosauriscus koeneni
Olenekian	Germany	10.12	51.59	Proterosuchia
Olenekian	Germany	11.71	51.79	Parasuchus
Olenekian	Morocco	-9.09	30.83	Synaptichnium pseudosuchoides
Olenekian	Morocco	-9.09	30.83	Synaptichnium
Olenekian	Morocco	-9.09	30.83	Brachychirotherium
Olenekian	Morocco	-9.09	30.83	Isochirotherium gierlinskii
Olenekian	Morocco	-9.09	30.83	Chirotherium barthii
Olenekian	Morocco	-9.09	30.83	Rhynchosauroides
Olenekian	North China	96.53	41.95	Beishanodon youngi
Olenekian	Norway	17.59	78.26	Reptilia
Olenekian	Poland	19.63	50.13	Archosauriformes
Olenekian	Poland	21.2	50.96	Brachychirotherium hauboldi
Olenekian	Poland	21.2	50.96	Brachychirotherium kalkowensis
Olenekian	Poland	21.2	50.96	Brachychirotherium wiorense
Olenekian	Poland	19.63	50.13	Collilongus rarus
Olenekian	Poland	19.63	50.13	Czatkowiella harae
Olenekian	Poland	21.2	50.96	Diapsida
Olenekian	Poland	19.04	50.39	Hemilopas mentzeli
Olenekian	Poland	21.2	50.96	Isochirotherium gierlinskii
Olenekian	Poland	21.2	50.96	Isochirotherium sanctacrucense
Olenekian	Poland	19.63	50.13	Osmolskina czatkowicensis
Olenekian	Poland	19.63	50.13	Pamelina polonica

Olenekian	Poland	19.63	50.13	Procolina teresae
Olenekian	Poland	21.2	50.96	Procolophonichnium polonicum
Olenekian	Poland	19.63	50.13	Procolophonidae
Olenekian	Poland	21.32	50.87	Prorotodactylus
Olenekian	Poland	21.2	50.96	Prorotodactylus mirus
Olenekian	Poland	21.2	50.96	Rhynchosauroides brevidigitatus
Olenekian	Poland	21.2	50.96	Rhynchosauroides rdzaneki
Olenekian	Poland	19.63	50.13	Sophineta cracoviensis
Olenekian	Poland	21.2	50.96	Synapsida
Olenekian	Poland	21.2	50.96	Synaptichnium chirotherioides
Olenekian	Poland	21.2	50.96	Synaptichnium kotanskii
Olenekian	Poland	21.2	50.96	Synaptichnium senkowiczowae
Olenekian	Russia	52.66	52.35	Archosauria
Olenekian	Russia	53.9	51.61	Archosauria
Olenekian	Russia	53.09	52.29	Archosauromorpha
Olenekian	Russia	55.89	51.28	Archosauromorpha
Olenekian	Russia	55.24	50.81	Archosauromorpha
Olenekian	Russia	62.72	68.62	Augustaburiania vatagini
Olenekian	Russia	43.67	49.26	Augustaburiania vatagini
Olenekian	Russia	49.93	69.28	Boreopricea
Olenekian	Russia	49.93	69.28	Boreopricea funerea
Olenekian	Russia	43.67	49.26	Bystrowisuchus flerovi
Olenekian	Russia	48.58	60.42	Chasmatosuchus
Olenekian	Russia	51.07	52.5	Chasmatosuchus
Olenekian	Russia	51.7	52.81	Chasmatosuchus
Olenekian	Russia	51.73	52.81	Chasmatosuchus
Olenekian	Russia	51.78	52.8	Chasmatosuchus
Olenekian	Russia	51.17	52.42	Chasmatosuchus
Olenekian	Russia	52.01	52.62	Chasmatosuchus
Olenekian	Russia	51.93	52.5	Chasmatosuchus
Olenekian	Russia	52.35	52.83	Chasmatosuchus
Olenekian	Russia	52.36	52.54	Chasmatosuchus
Olenekian	Russia	52.66	52.35	Chasmatosuchus
Olenekian	Russia	52.69	52.37	Chasmatosuchus
Olenekian	Russia	53.08	52.3	Chasmatosuchus
Olenekian	Russia	53.05	52.21	Chasmatosuchus
Olenekian	Russia	53.08	52.21	Chasmatosuchus
Olenekian	Russia	53.33	52.22	Chasmatosuchus
Olenekian	Russia	53.64	51.89	Chasmatosuchus
Olenekian	Russia	55.2	51.45	Chasmatosuchus
Olenekian	Russia	49.56	62.06	Chasmatosuchus magnus
Olenekian	Russia	55.08	51.75	Chasmatosuchus magnus
Olenekian	Russia	45.17	59.78	Chasmatosuchus rossicus
Olenekian	Russia	45.5	59.72	Chasmatosuchus rossicus
Olenekian	Russia	51.77	52.81	Chasmatosuchus rossicus
Olenekian	Russia	43.67	49.26	Coelodontognathus donensis
Olenekian	Russia	43.67	49.26	Coelodontognathus ricovi
Olenekian	Russia	52.11	65.45	Crocopoda

Olenekian	Russia	52.17	65.42	Crocopoda
Olenekian	Russia	57.37	65.02	Crocopoda
Olenekian	Russia	45.17	59.78	Crocopoda
Olenekian	Russia	50.18	59.5	Crocopoda
Olenekian	Russia	43.67	49.26	Crocopoda
Olenekian	Russia	51.17	52.42	Crocopoda
Olenekian	Russia	52.3	52.38	Crocopoda
Olenekian	Russia	53.09	52.29	Crocopoda
Olenekian	Russia	55.24	50.81	Galesauridae
Olenekian	Russia	55.53	52.4	Garjainia
Olenekian	Russia	55.76	52.43	Garjainia
Olenekian	Russia	55.84	52.15	Garjainia
Olenekian	Russia	55.85	52.16	Garjainia
Olenekian	Russia	55.65	52.03	Garjainia
Olenekian	Russia	55.89	51.28	Garjainia
Olenekian	Russia	55.72	52.03	Garjainia
Olenekian	Russia	55.2	51.45	Garjainia
Olenekian	Russia	55.08	51.75	Garjainia prima
Olenekian	Russia	55.24	50.81	Garjainia prima
Olenekian	Russia	43.67	49.26	Kapes
Olenekian	Russia	49.56	62.06	Kapes amaenus
Olenekian	Russia	49.56	62.06	Kapes komiensis
Olenekian	Russia	55.64	52.03	Kapes majmesculae
Olenekian	Russia	51.7	52.81	Microcnemus
Olenekian	Russia	45.17	59.78	Microcnemus efremovi
Olenekian	Russia	45.5	59.72	Microcnemus efremovi
Olenekian	Russia	49.93	69.28	Orenburgia
Olenekian	Russia	56.01	75.18	Orenburgia bruma
Olenekian	Russia	62.72	68.62	Orenburgia bruma
Olenekian	Russia	49.93	69.28	Orenburgia concinna
Olenekian	Russia	62.72	68.62	Orenburgia concinna
Olenekian	Russia	52.66	52.35	Orenburgia concinna
Olenekian	Russia	43.67	49.26	Orenburgia enigmaticus
Olenekian	Russia	49.93	69.28	Procolophonoidea
Olenekian	Russia	45.17	59.78	Procolophonoidea
Olenekian	Russia	49.93	69.28	Prolacertidae
Olenekian	Russia	43.67	49.26	Putillosaurus sennikovi
Olenekian	Russia	50.87	52.51	Reptilia
Olenekian	Russia	51.33	52.72	Reptilia
Olenekian	Russia	52.08	52.86	Reptilia
Olenekian	Russia	52.22	52.49	Reptilia
Olenekian	Russia	53.11	52.31	Reptilia
Olenekian	Russia	53.8	51.61	Reptilia
Olenekian	Russia	53.86	51.59	Reptilia
Olenekian	Russia	54.31	51.3	Reptilia
Olenekian	Russia	51.73	52.86	Rhynchocephalia
Olenekian	Russia	51.78	52.8	Scharschengia
Olenekian	Russia	43.67	49.26	Scythosuchus basileus

Olenekian	Russia	55.08	51.75	Silphedosuchus orenburgensis
Olenekian	Russia	55.89	52.15	Thecodontia
Olenekian	Russia	51.73	52.81	Tichvinskia
Olenekian	Russia	53.34	52.22	Tichvinskia
Olenekian	Russia	55.28	51.39	Tichvinskia burtensis
Olenekian	Russia	55.24	50.81	Tichvinskia burtensis
Olenekian	Russia	50.18	59.5	Tichvinskia vjatkensis
Olenekian	Russia	48.05	64.56	Timanophon raridentatus
Olenekian	Russia	48.78	64.56	Timanophon raridentatus
Olenekian	Russia	52.11	65.45	Timanophon raridentatus
Olenekian	Russia	43.67	49.26	Vitalia grata
Olenekian	Russia	55.08	51.75	Vritramimosaurus dzerzhinskii
Olenekian	Russia	49.56	62.06	Vytshegdosuchus zheshartensis
Olenekian	South Africa	27.96	-28.18	Archosauriformes
Olenekian	South Africa	26.12	-32.2	Coletta seca
Olenekian	South Africa	27.96	-28.18	Eucynodontia
Olenekian	South Africa	27.81	-28.46	Garjainia
Olenekian	South Africa	27.62	-28.32	Garjainia madiba
Olenekian	South Africa	27.7	-28.3	Garjainia madiba
Olenekian	South Africa	27.82	-28.46	Garjainia madiba
Olenekian	South Africa	27.82	-28.46	Garjainia madiba
Olenekian	South Africa	27.96	-28.18	Garjainia madiba
Olenekian	South Africa	27.97	-28.23	Garjainia madiba
Olenekian	South Africa	28.7	-28.32	Garjainia madiba
Olenekian	South Africa	27.1	-32.29	Kitchingnathus untabeni
Olenekian	South Africa	26.25	-32.01	Langbergia modisei
Olenekian	South Africa	26.84	-30.42	Langbergia modisei
Olenekian	South Africa	26.84	-30.42	Langbergia modisei
Olenekian	South Africa	27.43	-28.67	Langbergia modisei
Olenekian	South Africa	27.62	-28.32	Langbergia modisei
Olenekian	South Africa	27.95	-28.3	Langbergia modisei
Olenekian	South Africa	27.96	-28.18	Langbergia modisei
Olenekian	South Africa	27.97	-28.23	Langbergia modisei
Olenekian	South Africa	28.3	-28.2	Langbergia modisei
Olenekian	South Africa	28.7	-28.32	Langbergia modisei
Olenekian	South Africa	27.1	-32.29	Lystrosaurus
Olenekian	South Africa	26.84	-30.42	Microgomphodon oligocynus
Olenekian	South Africa	28.7	-28.32	Microgomphodon oligocynus
Olenekian	South Africa	27.96	-28.18	Palacrodon browni
Olenekian	South Africa	26.37	-30.59	Procolophon
Olenekian	South Africa	27.1	-32.29	Procolophon trigoniceps
Olenekian	South Africa	27.96	-28.18	Procolophonidae
Olenekian	South China	117.82	31.62	Cartorhynchus lenticarpus
Olenekian	South China	111.56	31.22	Eohupehsuchus brevicollis
Olenekian	South China	111.57	31.16	Hupehsuchus nanchangensis
Olenekian	South China	111.64	31.06	Nanchangosaurus suni
Olenekian	South China	111.64	31.06	Parahupehsuchus longus
Olenekian	South China	117.82	31.62	Sclerocormus parviceps

Olenekian	UK	-2.18	52.39	Aetosauripus
Olenekian	UK	-2.18	52.39	Reptilia
Olenekian	UK	-2.18	52.39	Coelurosaurichnus ziegelangernensis
Olenekian	UK	-2.18	52.39	Coelurosaurichnus
Olenekian	USA	-111	35.29	Archosauromorpha
Olenekian	USA	-108.03	43.67	Chelonipus
Olenekian	USA	-107.18	43.28	Chelonipus
Olenekian	USA	-107.12	43.33	Chelonipus
Olenekian	USA	-113.13	37.43	Chirotherium
Olenekian	USA	-108.03	43.67	Chirotherium barthii
Olenekian	USA	-107.18	43.28	Chirotherium barthii
Olenekian	USA	-107.12	43.33	Chirotherium barthii
Olenekian	USA	-106.93	43.42	Chirotherium barthii
Olenekian	USA	-111.03	35.04	Chirotherium rex
Olenekian	USA	-110.87	35.07	Chirotherium rex
Olenekian	USA	-111.03	35.04	Chirotherium sickleri
Olenekian	USA	-113.07	37.17	Eubrontes
Olenekian	USA	-113.07	37.17	Grallator
Olenekian	USA	-111.03	35.04	Isochirotherium coltoni
Olenekian	USA	-111.03	35.04	Procolophonichnium
Olenekian	USA	-113.07	37.17	Reptilia
Olenekian	USA	-111.03	35.04	Reptilia
Olenekian	USA	-113.13	37.43	Rhynchosauroides
Olenekian	USA	-111.03	35.04	Rhynchosauroides
Olenekian	USA	-110.43	37.81	Rhynchosauroides
Olenekian	USA	-108.03	43.67	Rhynchosauroides
Olenekian	USA	-107.18	43.28	Rhynchosauroides
Olenekian	USA	-107.12	43.33	Rhynchosauroides
Olenekian	USA	-106.93	43.42	Rhynchosauroides
Olenekian	USA	-107.8	43.47	Rhynchosauroides pallinii
Olenekian	USA	-107.18	43.28	Rotodactylus
Olenekian	USA	-111.03	35.04	Rotodactylus cursorius
Olenekian	USA	-111.03	35.04	Synaptichnium diabloensis
Olenekian	USA	-113.07	37.17	Therapsida
Anisian	Algeria	4.17	36.46	Rotodactylus bessieri
Anisian	Antarctica	164.35	-84.28	Angonisaurus
Anisian	Antarctica	164.35	-84.28	Archosauria
Anisian	Antarctica	164.05	-84.35	Cynognathidae
Anisian	Antarctica	164.05	-84.35	Cynognathus
Anisian	Antarctica	164.05	-84.35	Diademodon
Anisian	Antarctica	164.05	-84.35	Kannemeyeriiformes
Anisian	Antarctica	164.35	-84.28	Therocephalia
Anisian	Argentina	-69.25	-32.95	Andescynodon mendozensis
Anisian	Argentina	-69.25	-32.95	Vinceria andina
Anisian	Argentina	-69.2	-32.95	Cromptodon mamiferoides
Anisian	China-Xinjiang	89.17	42.92	Turfanosuchus dabanensis
Anisian	China-Xinjiang	89.17	42.92	Youngosuchus sinensis
Anisian	China-Xinjiang	89.17	42.92	Xiyukannemeyeria brevirostris

Anisian	France	3.36	43.75	Brachychirotherium circaparvum
Anisian	France	7.24	48.81	Chirotheriidae
Anisian	France	7.24	48.86	Chirotheriidae
Anisian	France	7.28	48.88	Chirotheriidae
Anisian	France	7.18	48.72	Chirotherium
Anisian	France	3.36	43.75	Chirotherium barthii
Anisian	France	3.36	43.75	Chirotherium ferox
Anisian	France	3.36	43.75	Prorotodactylus lutevensis
Anisian	France	6.86	49.15	Reptilia
Anisian	France	3.36	43.75	Rhynchosauroides
Anisian	France	7.25	48.82	Rhynchosauroides petri
Anisian	France	3.36	43.75	Rotodactylus bessieri
Anisian	Germany	8.59	48.54	Amotosaurus rotfeldensis
Anisian	Germany	8.7	48.61	Amotosaurus rotfeldensis
Anisian	Germany	8.16	47.68	Amotosaurus rotfeldensis
Anisian	Germany	8.59	48.54	Anomoiodon krejcii
Anisian	Germany	10.65	50.45	Anomoiodon liliensterni
Anisian	Germany	8.16	47.68	Ctenosauriscidae
Anisian	Germany	6.63	50.29	Eifelosaurus triadicus
Anisian	Germany	11.03	50.33	Koiloskiosaurus coburgiensis
Anisian	Germany	8.34	47.82	Protanystropheus antiquus
Anisian	Germany	7.77	47.56	Sclerosaurus armatus
Anisian	Germany	8.42	48.45	Thecodontia
Anisian	Germany	8.59	48.54	Thecodontia
Anisian	Germany	11.64	49.96	Thecodontosaurus
Anisian	Germany	11.77	51.22	Theropoda
Anisian	Germany	11.73	51.8	Trachelosaurus fischeri
Anisian	India	78.57	22.6	Dicynodontia
Anisian	India	79.25	19.38	Mesodapedon kuttyi
Anisian	India	79.68	18.86	Pamelaria dolichotrachela
Anisian	India	79.7	18.83	Rechnisaurus cristarhynchus
Anisian	India	78.57	22.6	Rhynchosauria
Anisian	India	78.57	22.63	Rhynchosauria
Anisian	India	78.48	22.62	Shringasaurus indicus
Anisian	India	79.7	18.83	Wadiasaurus indicus
Anisian	India	79.68	18.86	Yarasuchus deccanensis
Anisian	Israel	34.79	30.35	Tanystropheus haasi
Anisian	Israel	34.88	30.57	Tanystropheus
Anisian	Israel	34.88	30.57	Reptilia
Anisian	Italy	11.11	46.53	Brachychirotherium
Anisian	Italy	12	46.66	Brachychirotherium
Anisian	Italy	12	46.66	Chirotherium
Anisian	Italy	11.11	46.53	Chirotherium barthii
Anisian	Italy	12	46.66	Chirotherium ladinicus
Anisian	Italy	11.97	46.7	Chirotherium parvum
Anisian	Italy	11.97	46.7	Chirotherium rex
Anisian	Italy	11.11	46.53	Dinosauromorpha
Anisian	Italy	8.81	45.87	Helveticosaurus zollingeri

Anisian	Italy	13.2	46.49	Heteropelta boboi
Anisian	Italy	12	46.66	Isochirotherium
Anisian	Italy	11.11	46.53	Isochirotherium delicatum
Anisian	Italy	11.97	46.7	Isochirotherium delicatum
Anisian	Italy	12.12	46.74	Megachirella wachtleri
Anisian	Italy	11.11	46.53	Parasynaptichnium
Anisian	Italy	12	46.66	Procolophonichnium
Anisian	Italy	11.11	46.53	Rhynchosauroides
Anisian	Italy	12	46.66	Rhynchosauroides
Anisian	Italy	11.97	46.7	Rhynchosauroides tirolicus
Anisian	Italy	11.11	46.53	Rotodactylus
Anisian	Italy	12	46.66	Rotodactylus
Anisian	Italy	11.11	46.53	Synaptichnium
Anisian	Italy	8.81	45.86	Ticinosuchus
Anisian	Italy	8.91	45.89	Tribelesodon longobardicus
Anisian	Malawi	34.57	-10.52	Archosauriformes
Anisian	Malawi	34.57	-10.52	Anomodontia
Anisian	Morocco	-9.09	30.82	Atreipus
Anisian	Morocco	-9.09	30.81	Chirotheriidae
Anisian	Morocco	-9.09	30.82	Chirotherium barthii
Anisian	Morocco	-9.09	30.82	Chirotherium barthii
Anisian	Morocco	-9.09	30.83	Isochirotherium coureli
Anisian	Morocco	-9.09	30.82	Isochirotherium coureli
Anisian	Morocco	-9.09	30.82	Procolophonichnium
Anisian	Morocco	-9.09	30.82	Rhynchosauroides
Anisian	Morocco	-9.09	30.83	Rotodactylus
Anisian	Morocco	-9.09	30.83	Synaptichnium
Anisian	Morocco	-9.09	30.82	Synaptichnium
Anisian	Namibia	16.48	-21.1	Cynodontia
Anisian	Namibia	16.47	-21.1	Cynognathus
Anisian	Namibia	16.48	-21.1	Cynognathus
Anisian	Namibia	16.39	-21.06	Diademodon
Anisian	Namibia	16.47	-21.1	Diademodon tetragonus
Anisian	Namibia	16.45	-21.1	Dolichuranus primaevus
Anisian	Namibia	16.47	-21.1	Dolichuranus primaevus
Anisian	Namibia	16.43	-21.09	Etjoia dentitransitus
Anisian	Namibia	16.45	-21.1	Kannemeyeria lophorhinus
Anisian	Namibia	16.39	-21.06	Kannemeyeriidae
Anisian	Netherlands	6.73	51.97	Amotosaurus rotfeldensis
Anisian	Netherlands	6.73	51.97	Brachychirotherium paraparvum
Anisian	Netherlands	6.73	51.97	Chirotherium peabodyi
Anisian	Netherlands	6.73	51.97	Coelurosaurichnus ratumensis
Anisian	Netherlands	6.73	51.97	Eusaurosphargis
Anisian	Netherlands	6.78	51.97	Eusaurosphargis
Anisian	Netherlands	6.73	51.97	Procolophonichnium
Anisian	Netherlands	6.73	51.97	Procolophonichnium winterswijkense
Anisian	Netherlands	6.73	51.97	Rhynchosauroides
Anisian	Netherlands	6.73	51.97	Sustenodactylus hollandicus

Anisian	North China	112.85	36.97	Archosauriformes
Anisian	North China	112.9	36.92	Archosauriformes
Anisian	North China	110.7	37.29	Archosauromorpha
Anisian	North China	110.97	39.68	Eumetabolodon bathycephalus
Anisian	North China	110.97	39.68	Euparkeriidae
Anisian	North China	111	39.1	Guchengosuchus shiguaiensis
Anisian	North China	111	39.1	Halazhaisuchus qiaoensis
Anisian	North China	110.7	37.29	Kannemeyeriidae
Anisian	North China	112.3	39	Kannemeyeriidae
Anisian	North China	112.3	39	Kannemeyeriidae
Anisian	North China	112.85	36.97	Kannemeyeriidae
Anisian	North China	112.88	36.98	Kannemeyeriidae
Anisian	North China	112.9	36.92	Kannemeyeriidae
Anisian	North China	113	37.08	Neoprocolophon asiaticus
Anisian	North China	110.7	37.29	Nothogomphodon sanjiaoensis
Anisian	North China	110.99	38.82	Ordosiodon lincheyuensis
Anisian	North China	110.97	39.68	Ordosiodon youngi
Anisian	North China	112.3	39	Parakannemeyeria
Anisian	North China	112.85	36.97	Parakannemeyeria
Anisian	North China	112.88	36.93	Parakannemeyeria
Anisian	North China	112.3	39	Parakannemeyeria dolichocephala
Anisian	North China	111.13	38.47	Parakannemeyeria ningwuensis
Anisian	North China	112.3	39	Parakannemeyeria ningwuensis
Anisian	North China	110.75	38.48	Parakannemeyeria shenmuensis
Anisian	North China	112.85	36.97	Parakannemeyeria youngi
Anisian	North China	112.87	36.98	Parakannemeyeria youngi
Anisian	North China	110.99	38.82	Procolophonidae
Anisian	North China	112.87	36.98	Pseudosuchia
Anisian	North China	110.97	39.68	Shaanbeikannemeyeria buerdongia
Anisian	North China	110.85	39.43	Shaanbeikannemeyeria xilougouensis
Anisian	North China	112.85	36.97	Shansiodon
Anisian	North China	112.87	36.92	Shansiodon
Anisian	North China	113	37.08	Shansiodon
Anisian	North China	113.02	37.07	Shansiodon wangi
Anisian	North China	110.8	38.47	Shansiodon wuhsiangensis
Anisian	North China	112.87	36.98	Shansiodon wuhsiangensis
Anisian	North China	112.9	36.92	Shansiodon wuhsiangensis
Anisian	North China	110.8	38.47	Shansisuchus kuyeheensis
Anisian	North China	110.44	36.15	Shansisuchus shansisuchus
Anisian	North China	112.3	39	Shansisuchus shansisuchus
Anisian	North China	112.85	36.97	Shansisuchus shansisuchus
Anisian	North China	112.88	36.98	Shansisuchus shansisuchus
Anisian	North China	112.88	36.93	Shansisuchus shansisuchus
Anisian	North China	112.9	36.92	Shansisuchus shansisuchus
Anisian	North China	112.92	36.92	Shansisuchus shansisuchus
Anisian	North China	112.93	36.97	Shansisuchus shansisuchus
Anisian	North China	113.02	37.07	Shansisuchus shansisuchus
Anisian	North China	112.9	36.92	Sinognathus gracilis

Anisian	North China	112.87	36.98	Sinokannemeyeria
Anisian	North China	112.92	36.9	Sinokannemeyeria pearsoni
Anisian	North China	112.93	36.97	Sinokannemeyeria pearsoni
Anisian	North China	111.15	37.5	Sinokannemeyeria sanchuanheensis
Anisian	North China	113	37.08	Sinokannemeyeria yingchiaoensis
Anisian	North China	112.3	39	Thecodontia
Anisian	North China	112.85	36.97	Thecodontia
Anisian	North China	112.87	36.98	Thecodontia
Anisian	North China	112.9	36.92	Thecodontia
Anisian	North China	112.92	36.92	Thecodontia
Anisian	North China	113.02	37.07	Thecodontia
Anisian	North China	112.17	35.2	Traversodontoides wangwuensis
Anisian	North China	110.97	39.68	Yikezhaogia megafenestrala
Anisian	Poland	20.87	51.03	Brachychirotherium
Anisian	Poland	20.87	51.03	Chirotheriidae
Anisian	Poland	20.87	51.03	Chirotherium
Anisian	Poland	20.87	51.03	Chirotherium barthii
Anisian	Poland	18.97	50.3	Cladeiodon
Anisian	Poland	17.63	50.58	Hemilopas mentzeli
Anisian	Poland	18.08	50.48	Hemilopas mentzeli
Anisian	Poland	18.95	50.49	Hemilopas mentzeli
Anisian	Poland	18.97	50.3	Hemilopas mentzeli
Anisian	Poland	20.87	51.03	Isochirotherium herculis
Anisian	Poland	20.87	51.03	Isochirotherium soergeli
Anisian	Poland	17.63	50.58	Protanystropheus antiquus
Anisian	Poland	17.97	50.47	Protanystropheus antiquus
Anisian	Poland	17.63	50.58	Protorosauridae
Anisian	Poland	20.87	51.03	Rhynchosauroides bornemanni
Anisian	Poland	20.87	51.03	Rhynchosauroides pallinii
Anisian	Poland	20.87	51.03	Rotodactylus
Anisian	Poland	20.87	51.03	Synaptichnium
Anisian	Poland	17.63	50.58	Theropoda
Anisian	Romania	22.35	47.1	Tanystropheus biharicus
Anisian	Russia	55.49	51.36	Antecosuchus boreus
Anisian	Russia	55.36	51.55	Antecosuchus ochevi
Anisian	Russia	55.43	51.42	Antecosuchus ochevi
Anisian	Russia	55.65	52.41	Archosauria
Anisian	Russia	55.31	51.36	Bauriidae
Anisian	Russia	55.65	52.41	Dicynodontia
Anisian	Russia	53.5	52.53	Dongusuchus efremovi
Anisian	Russia	55.18	51.46	Dongusuchus efremovi
Anisian	Russia	55.31	51.36	Dongusuchus efremovi
Anisian	Russia	55.31	51.36	Dorosuchus neoetus
Anisian	Russia	55.43	51.42	Dorosuchus neoetus
Anisian	Russia	53.5	52.53	Jushatyria vjushkovi
Anisian	Russia	53.5	52.53	Kannemeyeriiformes
Anisian	Russia	55.65	52.41	Kannemeyeriiformes
Anisian	Russia	55.43	51.42	Kapes majmesculae

Anisian	Russia	55.36	51.55	Nothogomphodon danilovi
Anisian	Russia	55.31	51.36	Pseudosuchia
Anisian	Russia	53.5	52.53	Rabidosaurus cristatus
Anisian	Russia	55.31	51.36	Rabidosaurus cristatus
Anisian	Russia	53.5	52.53	Rhadiodromus klimovi
Anisian	Russia	55.31	51.36	Rhadiodromus klimovi
Anisian	Russia	55.43	51.42	Rhadiodromus mariae
Anisian	Russia	55.43	51.42	Rhinodicynodon gracile
Anisian	Russia	55.36	51.55	Sarmatosuchus otschevi
Anisian	Russia	55.31	51.36	Thecodontia
Anisian	Russia	55.49	51.36	Uralokannemeyeria vjuschkovi
Anisian	Russia	53.5	52.53	Uralosaurus magnus
Anisian	Russia	55.31	51.36	Uralosaurus magnus
Anisian	Russia	55.49	51.36	Uralosaurus magnus
Anisian	Russia	53.5	52.53	Vjushkovisaurus berdjanensis
Anisian	Russia	55.36	51.55	Vjushkovisaurus berdjanensis
Anisian	South Africa	26.7	-30.68	Aelurosuchus browni
Anisian	South Africa	27.23	-31.7	Bauria cynops
Anisian	South Africa	27.23	-31.7	Bauria cynops
Anisian	South Africa	27.23	-31.7	Bauria cynops
Anisian	South Africa	27.24	-31.71	Bauria cynops
Anisian	South Africa	26.33	-31.07	Bauria cynops
Anisian	South Africa	26.33	-31	Bauria cynops
Anisian	South Africa	26.33	-30.99	Bauria cynops
Anisian	South Africa	26.11	-30.91	Bauria cynops
Anisian	South Africa	26.27	-30.42	Bauria cynops
Anisian	South Africa	26.33	-31	Bauria robusta
Anisian	South Africa	27.23	-31.7	Bolotridon frerensis
Anisian	South Africa	26.28	-30.77	Cistecynodon parvus
Anisian	South Africa	26.33	-31	Cricodon kannemeyeri
Anisian	South Africa	26.38	-31.6	Cricodon metabolus
Anisian	South Africa	26.38	-31.61	Cricodon metabolus
Anisian	South Africa	26.38	-31.6	Cynodontia
Anisian	South Africa	26.38	-31.6	Cynognathus
Anisian	South Africa	26.32	-30.99	Cynognathus
Anisian	South Africa	27.23	-31.7	Cynognathus crateronotus
Anisian	South Africa	27.23	-31.7	Cynognathus crateronotus
Anisian	South Africa	27.23	-31.7	Cynognathus crateronotus
Anisian	South Africa	27.24	-31.71	Cynognathus crateronotus
Anisian	South Africa	26.33	-31	Cynognathus crateronotus
Anisian	South Africa	26.11	-30.91	Cynognathus crateronotus
Anisian	South Africa	26.7	-30.68	Cynognathus crateronotus
Anisian	South Africa	26.38	-31.6	Diademodon
Anisian	South Africa	26.38	-31.61	Diademodon
Anisian	South Africa	27.23	-31.7	Diademodon
Anisian	South Africa	26.33	-31.07	Diademodon
Anisian	South Africa	26.33	-31	Diademodon
Anisian	South Africa	26.32	-30.99	Diademodon

Anisian	South Africa	27.9	-31.73	Diademodon
Anisian	South Africa	27.24	-31.71	Diademodon browni
Anisian	South Africa	26.94	-31.78	Diademodon tetragonus
Anisian	South Africa	27.23	-31.7	Diademodon tetragonus
Anisian	South Africa	26.33	-31	Diademodon tetragonus
Anisian	South Africa	26.11	-30.91	Diademodon tetragonus
Anisian	South Africa	26.62	-30.61	Eohyosaurus wolvaardti
Anisian	South Africa	27.23	-31.7	Erythrosuchus africanus
Anisian	South Africa	27.24	-31.71	Erythrosuchus africanus
Anisian	South Africa	26.11	-30.91	Erythrosuchus africanus
Anisian	South Africa	26.62	-30.61	Erythrosuchus africanus
Anisian	South Africa	26.97	-30.83	Erythrosuchus africanus
Anisian	South Africa	26.79	-30.7	Erythrosuchus africanus
Anisian	South Africa	26.71	-30.69	Euparkeria capensis
Anisian	South Africa	26.7	-30.68	Howesia browni
Anisian	South Africa	26.32	-30.99	Kannemeyeria
Anisian	South Africa	26.22	-30.88	Kannemeyeria
Anisian	South Africa	26.49	-30.66	Kannemeyeria
Anisian	South Africa	26.62	-30.61	Kannemeyeria
Anisian	South Africa	24.98	-31.65	Kannemeyeria simocephala
Anisian	South Africa	26.94	-31.78	Kannemeyeria simocephala
Anisian	South Africa	27.23	-31.7	Kannemeyeria simocephala
Anisian	South Africa	27.23	-31.7	Kannemeyeria simocephala
Anisian	South Africa	27.23	-31.7	Kannemeyeria simocephala
Anisian	South Africa	26.33	-31	Kannemeyeria simocephala
Anisian	South Africa	26.11	-30.91	Kannemeyeria simocephala
Anisian	South Africa	27.9	-31.73	Kannemeyeriidae
Anisian	South Africa	26.37	-31.61	Kannemeyeriiformes
Anisian	South Africa	26.37	-31.6	Kannemeyeriiformes
Anisian	South Africa	26.39	-31.54	Kannemeyeriiformes
Anisian	South Africa	26.4	-31.58	Kannemeyeriiformes
Anisian	South Africa	27.23	-31.7	Kombuisia frerensis
Anisian	South Africa	27.24	-31.71	Lumkuia fuzzi
Anisian	South Africa	26.71	-30.69	Mesosuchus browni
Anisian	South Africa	27.23	-31.7	Microgomphodon oligocynus
Anisian	South Africa	26.7	-30.68	Microgomphodon oligocynus
Anisian	South Africa	26.62	-30.61	Microgomphodon oligocynus
Anisian	South Africa	26.83	-30.42	Microgomphodon oligocynus
Anisian	South Africa	26.33	-31	Microhelodon eumerus
Anisian	South Africa	26.7	-30.68	Nythosaurus browni
Anisian	South Africa	26.7	-30.68	Palacrodon browni
Anisian	South Africa	26.7	-30.68	Procolophonidae
Anisian	South Africa	26.62	-30.61	Procolophonidae
Anisian	South Africa	26.7	-30.68	Procolophoninae
Anisian	South Africa	27.24	-31.71	Protacmon reubsameni
Anisian	South Africa	27.24	-31.71	Sesamondontoides pauli
Anisian	South Africa	26.38	-31.61	Shansiodon
Anisian	South Africa	27.24	-31.71	Sysphinctostoma gracilis

Anisian	South Africa	27.23	-31.7	Sysphinctostoma smithi
Anisian	South Africa	26.61	-30.62	Teratophon spinigenis
Anisian	South Africa	26.83	-30.42	Theledectes perforatus
Anisian	South Africa	26.11	-30.91	Thelephon contritus
Anisian	South Africa	26.83	-30.42	Thelerpeton oppressus
Anisian	South Africa	26.62	-30.61	Trirachodon
Anisian	South Africa	27.23	-31.7	Trirachodon berryi
Anisian	South Africa	26.33	-31	Trirachodon berryi
Anisian	South Africa	26.11	-30.91	Trirachodon berryi
Anisian	South Africa	26.7	-30.68	Trirachodon minor
Anisian	South Africa	26.39	-31.61	Ufudocyclops mukanelai
Anisian	South Africa	26.4	-31.53	Ufudocyclops mukanelai
Anisian	South Africa	26.5	-31.55	Ufudocyclops mukanelai
Anisian	South China	104.31	24.88	Largocephalosaurus polycarpon
Anisian	South China	104.47	25.7	Largocephalosaurus qianensis
Anisian	South China	111.69	31.16	Lotosaurus
Anisian	South China	110.3	29.5	Lotosaurus adentus
Anisian	South China	104.33	24.78	Pectodens zhenyuensis
Anisian	South China	104.47	25.7	Qianosuchus mixtus
Anisian	South China	104.33	24.73	Sinosaurosphargis yunguiensis
Anisian	South China	104.85	25.48	Sinosaurosphargis yunguiensis
Anisian	Spain	2.33	41.78	Archosauromorpha
Anisian	Spain	-1.63	39.98	Chirotherium
Anisian	Spain	-1.04	40.15	Chirotherium barthii
Anisian	Spain	-0.99	40.4	Chirotherium barthii
Anisian	Spain	-1.63	39.98	Coelurosaurichnus perriauxi
Anisian	Spain	-0.99	40.4	Isochirotherium coureli
Anisian	Spain	-1.63	39.98	Paratrisauropus latus
Anisian	Spain	2.3	41.77	Procolophonidae
Anisian	Spain	2.33	41.78	Procolophonoidea
Anisian	Spain	-0.99	40.4	Rhynchosauroides
Anisian	Spain	0.03	40.07	Rhynchosauroides
Anisian	Switzerland	7.65	47.58	Basileosaurus freyi
Anisian	Switzerland	8.94	45.91	Macrocnemus bassanii
Anisian	Switzerland	7.65	47.58	Sclerosaurus armatus
Anisian	Switzerland	8.94	45.91	Ticinosuchus ferox
Anisian	Switzerland	8.94	45.91	Ticinosuchus ferox
Anisian	Tanzania	34.78	-10.47	Aleodon brachyrhamphus
Anisian	Tanzania	35.22	-10.33	Angonisaurus cruickshanki
Anisian	Tanzania	35.13	-10.3	Archosauriformes
Anisian	Tanzania	35.27	-10.38	Archosauriformes
Anisian	Tanzania	35.5	-10.47	Archosauriformes
Anisian	Tanzania	35.31	-10.3	Archosauromorpha
Anisian	Tanzania	35.13	-10.3	Asilisaurus kongwe
Anisian	Tanzania	35.13	-10.3	Asilisaurus kongwe
Anisian	Tanzania	35.13	-10.3	Asilisaurus kongwe
Anisian	Tanzania	35.34	-10.13	Asilisaurus kongwe
Anisian	Tanzania	35.24	-10.36	Asperoris mnyama

Anisian	Tanzania	35.13	-10.3	Cricodon metabolus
Anisian	Tanzania	35.16	-10.49	Cricodon metabolus
Anisian	Tanzania	35.27	-10.38	Cricodon metabolus
Anisian	Tanzania	35.13	-10.3	Cynodontia
Anisian	Tanzania	35.16	-10.38	Cynodontia
Anisian	Tanzania	35.25	-10.3	Cynognathus crateronotus
Anisian	Tanzania	34.78	-10.47	Diademodontidae
Anisian	Tanzania	35.23	-10.28	Dicynodontia
Anisian	Tanzania	35.24	-10.36	Dicynodontia
Anisian	Tanzania	35.22	-10.34	Hypselorhachis mirabilis
Anisian	Tanzania	34.84	-10.35	Kannemeyeria simocephala
Anisian	Tanzania	35.5	-10.47	Mambawakale ruhuhu
Anisian	Tanzania	35.22	-10.33	Mandagomphodon hirschsoni
Anisian	Tanzania	34.75	-10.51	Mandaphon nadra
Anisian	Tanzania	34.84	-10.35	Mandasuchus tanyauchen
Anisian	Tanzania	35.27	-10.38	Mandasuchus tanyauchen
Anisian	Tanzania	35.31	-10.3	Mandasuchus tanyauchen
Anisian	Tanzania	35.22	-10.34	Nundasuchus songeaensis
Anisian	Tanzania	34.75	-10.53	Nyasasaurus parringtoni
Anisian	Tanzania	35.23	-10.28	Nyasasaurus parringtoni
Anisian	Tanzania	35.23	-10.28	Parringtonia gracilis
Anisian	Tanzania	35.22	-10.34	Rechnisaurus cristarhynchus
Anisian	Tanzania	34.78	-10.47	Ruhuhuaria reiszi
Anisian	Tanzania	35.16	-10.38	Sangusaurus parringtonii
Anisian	Tanzania	35.22	-10.34	Sangusaurus parringtonii
Anisian	Tanzania	35.23	-10.28	Scalenodon
Anisian	Tanzania	34.78	-10.47	Scalenodon angustifrons
Anisian	Tanzania	35.16	-10.38	Scalenodon angustifrons
Anisian	Tanzania	35	-10.44	Scalenodon attridgei
Anisian	Tanzania	34.75	-10.53	Scalenodon charigi
Anisian	Tanzania	35.5	-10.47	Silesauridae
Anisian	Tanzania	34.78	-10.47	Stagonosuchus major
Anisian	Tanzania	35.27	-10.38	Stagonosuchus major
Anisian	Tanzania	35.25	-10.33	Stagonosuchus nyassicus
Anisian	Tanzania	35.27	-10.38	Stagonosuchus tanganyikaensis
Anisian	Tanzania	35.23	-10.28	Stenaulorhynchus
Anisian	Tanzania	35.31	-10.3	Stenaulorhynchus
Anisian	Tanzania	35.27	-10.38	Stenaulorhynchus stockleyi
Anisian	Tanzania	34.91	-10.36	Teleocrater rhadinus
Anisian	Tanzania	34.91	-10.36	Teleocrater rhadinus
Anisian	Tanzania	35.25	-10.3	Teleocrater rhadinus
Anisian	Tanzania	35.25	-10.33	Tetragonias njalilus
Anisian	Tanzania	35.31	-10.3	Tetragonias njalilus
Anisian	Tanzania	34.84	-10.35	Theriognathus microps
Anisian	Tanzania	35.25	-10.33	Traversodontidae
Anisian	UK	-3.27	50.67	Amniota
Anisian	UK	-3.27	50.66	Archosauria
Anisian	UK	-3.25	50.68	Archosauria

Anisian	UK	-3.23	50.68	Archosauria
Anisian	UK	-3.29	50.56	Archosauria
Anisian	UK	-1.58	52.29	Archosauria
Anisian	UK	-3.27	50.68	Bentonyx sidensis
Anisian	UK	-1.58	52.29	Bromsgroveia
Anisian	UK	-2.08	52.33	Bromsgroveia walkeri
Anisian	UK	-1.58	52.29	Bromsgroveia walkeri
Anisian	UK	-1.15	52.97	Chirotheriidae
Anisian	UK	-1.08	52.96	Chirotherioidea
Anisian	UK	-3.23	53.38	Chirotherium
Anisian	UK	-2.63	53.34	Chirotherium
Anisian	UK	-2.7	52.81	Chirotherium
Anisian	UK	-2.31	52.73	Chirotherium
Anisian	UK	-2.17	52.67	Chirotherium
Anisian	UK	-1.29	52.76	Chirotherium
Anisian	UK	-2.63	53.34	Chirotherium barthii
Anisian	UK	-2.43	53.38	Chirotherium barthii
Anisian	UK	-2.31	52.73	Chirotherium barthii
Anisian	UK	-2.31	52.73	Chirotherium sickleri
Anisian	UK	-2.43	53.38	Chirotherium storetonense
Anisian	UK	-2.17	52.67	Chirotherium storetonense
Anisian	UK	-1.15	52.97	Chirotherium swinnertoni
Anisian	UK	-2.43	53.38	Chirotherium vorbachi
Anisian	UK	-1.62	52.81	Chirotherium vorbachi
Anisian	UK	-3.23	50.68	Coartaredens isaaci
Anisian	UK	-1.35	52.95	Deuterotetrapous plancus
Anisian	UK	-3.26	50.67	Feralisaurus corami
Anisian	UK	-3.31	50.63	Fodonyx spenceri
Anisian	UK	-3.28	50.66	Fodonyx spenceri
Anisian	UK	-3.23	50.68	Fodonyx spenceri
Anisian	UK	-2.64	53.17	Isochirotherium
Anisian	UK	-2.7	52.81	Isochirotherium
Anisian	UK	-3.27	50.67	Kapes bentoni
Anisian	UK	-3.23	50.68	Kapes bentoni
Anisian	UK	-2.08	52.33	Langeronyx brodiei
Anisian	UK	-1.58	52.29	Langeronyx brodiei
Anisian	UK	-3.27	50.67	Parareptilia
Anisian	UK	-1.58	52.29	Phytosauria
Anisian	UK	-3.27	50.67	Procolophonidae
Anisian	UK	-2.08	52.33	Rhombopholis scutulata
Anisian	UK	-1.58	52.29	Rhombopholis scutulata
Anisian	UK	-3.28	50.66	Rhynchosauria
Anisian	UK	-1.58	52.29	Rhynchosauridae
Anisian	UK	-3.23	53.38	Rhynchosauroides
Anisian	UK	-2.7	52.81	Rhynchosauroides
Anisian	UK	-2.17	52.67	Rhynchosauroides
Anisian	UK	-2.7	52.81	Rhynchosauroides articeps
Anisian	UK	-3.23	53.38	Rhynchosauroides rectipes

Anisian	UK	-2.63	53.34	Rhynchosauroides rectipes
Anisian	UK	-1.58	52.29	Sauria
Anisian	UK	-1.58	52.29	Suchia
Anisian	UK	-2.17	52.67	Synaptichnium pseudosuchoides
Anisian	UK	-3.27	50.67	Tanystropheus
Anisian	UK	-1.15	52.97	Varanopus curvidactylus
Anisian	USA	-110.7	35.02	Ammorhynchus navajoi
Anisian	USA	-110.7	35.02	Ammorhynchus navajoi
Anisian	USA	-110.04	34.78	Ammorhynchus navajoi
Anisian	USA	-110.08	35.02	Ammorhynchus navajoi
Anisian	USA	-110.84	35.08	Anisodontosaurus greeri
Anisian	USA	-110.26	34.92	Anisodontosaurus greeri
Anisian	USA	-110.26	34.92	Archosauria
Anisian	USA	-105.14	35.2	Archosauriformes
Anisian	USA	-105.08	35.2	Archosauriformes
Anisian	USA	-110.84	35.08	Archosauromorpha
Anisian	USA	-105.08	35.2	Archosauromorpha
Anisian	USA	-111.4	35.87	Arizonasaurus babbitti
Anisian	USA	-110.84	35.08	Arizonasaurus babbitti
Anisian	USA	-110.5	35	Arizonasaurus babbitti
Anisian	USA	-110.3	34.94	Arizonasaurus babbitti
Anisian	USA	-110.26	34.92	Arizonasaurus babbitti
Anisian	USA	-105.08	35.2	Arizonasaurus babbitti
Anisian	USA	-110.26	34.92	Chirotherium
Anisian	USA	-111.47	35.84	Chirotherium barthii
Anisian	USA	-111.44	35.8	Chirotherium barthii
Anisian	USA	-110.07	34.5	Chirotherium barthii
Anisian	USA	-110.3	34.94	Chirotherium barthii
Anisian	USA	-113.04	37.17	Chirotherium rex
Anisian	USA	-111.44	35.8	Chirotherium rex
Anisian	USA	-110.3	34.94	Chirotherium rex
Anisian	USA	-110.84	35.08	Cynodontia
Anisian	USA	-105.08	35.2	Diapsida
Anisian	USA	-110.7	35.02	Dicynodontia
Anisian	USA	-110.15	34.9	Dicynodontia
Anisian	USA	-105.14	35.2	Dicynodontia
Anisian	USA	-110.25	34.92	Isochirotherium marshalli
Anisian	USA	-111.4	35.87	Poposauroidea
Anisian	USA	-110.84	35.08	Poposauroidea
Anisian	USA	-110.3	34.94	Poposauroidea
Anisian	USA	-110.26	34.92	Poposauroidea
Anisian	USA	-105.08	35.2	Poposauroidea
Anisian	USA	-105.08	35.2	Procolophonidae
Anisian	USA	-105.08	35.2	Pseudosuchia
Anisian	USA	-110.5	35	Reptilia
Anisian	USA	-110.3	34.94	Reptilia
Anisian	USA	-110.26	34.92	Reptilia
Anisian	USA	-110.15	34.9	Reptilia

Anisian	USA	-110.3	34.94	Rhynchosauroides
Anisian	USA	-111.48	35.91	Rhynchosauroides pallinii
Anisian	USA	-112	37.17	Rhynchosauroides pallinii
Anisian	USA	-111.4	35.87	Rotodactylus bradyi
Anisian	USA	-113.41	37.19	Rotodactylus cursorius
Anisian	USA	-111.47	35.84	Synaptichnium cameronensis
Anisian	USA	-110.15	34.9	Therapsipus cumminsi
Anisian	Zambia	32.99	-10.86	Cynognathus crateronotus
Anisian	Zambia	33.08	-10.75	Diademodon tetragonus
Anisian	Zambia	33.08	-10.75	Dolichuranus latirostris
Anisian	Zambia	33.08	-10.75	Kannemeyeria lophorhinus
Anisian	Zambia	33.08	-10.75	Kannemeyeriiformes
Anisian	Zambia	33.08	-10.75	Luangwa drysdalli
Anisian	Zambia	33.08	-10.75	Sangusaurus edentatus
Anisian	Zambia	33.08	-10.75	Trirachodon
Anisian	Zambia	33.08	-10.75	Zambiasaurus submersus

Table S4. Number of macro plant fossil extinct taxa and species level extinction magnitude.

	Changhsingian			Induan			Olenekian			Anisian		
	Total	High	Low	Total	High	Low	Total	High	Low	Total	High	Low
Taxa no.	352	151	245	166	112	72	241	104	158	289	132	181
No. extinct	288	100	217	109	74	43	166	79	92			
Extinction rate	82%	66%	86%	66%	66%	60%	69%	76%	58%			

Table S5. Fossil plant putative functional traits information for determing climate zone.

Age		Flora zone	Measurable plant fossils	Plant fossil recent location	Paleo latitude zone	Function related traits								Inferred climate zone	Inferred vegetation landscape baed on all the macro and micro fossil information
Period/series	stages					Plant form	Rough whole plant height (m)	Position in the flora	Pinna size (mm*2)		Vein type	Vein density (mm/mm*2)	Cuticle thickness		
									Compound leaf	Simple leaf					
End Permian	Changhsingian	Gondwana Flora	*Gangomopteris angustifolia*	Antarctic	High latitude	Tree	5–10	Canopy		1560	Pinnate net	1.92	Thin	Dsc	Tree dominant lowland forest
	Changhsingian	Gondwana Flora	*Glossopteris*	Antarctic	High latitude	Tree	10–30	Canopy		2960.4	Pinnate net		Thin		
	Changhsingian	Gondwana Flora	*Glossopteris arbeti*	Antarctic	High latitude	Tree	10–30	Canopy		3355.4	Pinnate net		Thin		
	Changhsingian	Gondwana Flora	*Glossopteris browniana*	Antarctic	High latitude	Tree	10–30	Canopy		591.5	Pinnate net	1.43	Thin		
	Changhsingian	Gondwana Flora	*Glossopteris bucklandensis*	Antarctic	High latitude	Tree	10–30	Canopy		4718.1	Pinnate net	2.06	Thin		
	Changhsingian	Gondwana Flora	*Glossopteris communis*	Antarctic	High latitude	Tree	10–30	Canopy		10323.7	Pinnate net		Thin		
	Changhsingian	Gondwana Flora	*Glossopteris longicaulis*	Antarctic	High latitude	Tree	10–30	Canopy		5471.4	Pinnate net		Thin		
	Changhsingian	Gondwana Flora	*Glossopteris major*	Antarctic	High latitude	Tree	10–30	Canopy		2178.3	Pinnate net		Thin		
	Changhsingian	Gondwana Flora	*Glossopteris taylori*	Antarctic	High latitude	Tree	10–30	Canopy		10634.6	Pinnate net		Thin		
	Changhsingian	Gondwana Flora	*Glossopteris tenuifolia*	Antarctic	High latitude	Tree	10–30	Canopy		1456.9	Pinnate net		Thin		
	Changhsingian	Gondwana Flora	*Sphenobaiera*	Argentina	Middle–high latitude	Tree	20–50	Canopy		1691.6	Parallel to simple net		Thick	Dsb	Tree dominant lowland and upland forest
	Changhsingian	Gondwana Flora	*Moltenia*	Argentina	Middle–high latitude	Tree to shrub		Canopy	1178.5		Pinnate to simple		Thick		
	Changhsingian	Gondwana Flora	*Pachydemophyllum*	Argentina	Middle–high latitude	Tree to shrub		Canopy	136.4		Pinnate to simple		Thick		
	Changhsingian	Gondwana Flora	*Zuberia*	Argentina	Middle–high latitude	Tree to shrub		Canopy	37.8		Pinnate to simple		Thick		
	Changhsingian	Gondwana Flora	*Voltziopsis africana*	Australia	High latitude	Tree–shrub	0.5–2	Canopy		2055.4	Simple		Thick	Dfb	Tree dominant upland forest
	Changhsingian	Gondwana Flora	*Glossopteris browniana*	Australia	High latitude	Tree	10–30	Canopy		39	Pinnate net		Thin		
	Changhsingian	Gondwana Flora	*Lepidopteris callipteroides*	Australia	High latitude	Tree to shrub	3–5	Canopy	5.9		Pinnate		Thick		
	Changhsingian	Cathaysian Flora	*Elatocladus conferta*	Jordan	Low latitude	Tree–shrub		Canopy		15.8	Simple	0.59	Thick	Cfb	Tree dominant lowland and upland forest
	Changhsingian	Cathaysian Flora	*Otovicia hypnoides*	Jordan	Low latitude	Tree–shrub		Canopy		1	Simple	1.70	Thick		
	Changhsingian	Cathaysian Flora	*Quadrocladus*	Jordan	Low latitude	Tree–shrub		Canopy		1.5	Simple		Thick		
	Changhsingian	Cathaysian Flora	*Quadrocladus*	Jordan	Low latitude	Tree–shrub		Canopy		6.8	Simple	1.27	Thick		
	Changhsingian	Cathaysian Flora	*Rissikia*	Jordan	Low latitude	Tree–shrub		Canopy		50.5	Simple	0.53	Thick		
	Changhsingian	Cathaysian Flora	*Rhipidopsis brevicaulis*	Jordan	Low latitude	Tree		Canopy		2920	Simple to simple net		Thick		
	Changhsingian	Cathaysian Flora	*Rhipidopsis panii*	Jordan	Low latitude	Tree		Canopy		32089.2	Simple to simple net		Thick		
	Changhsingian	Cathaysian Flora	*Sphenobaiera digitata*	Jordan	Low latitude	Tree	20–50	Canopy		5241.7	Simple to simple net		Thick		
	Changhsingian	Cathaysian Flora	*Dicrodium*	Jordan	Low latitude	Tree	5–10	Canopy	7.9		Pinnate		Thick		
	Changhsingian	Cathaysian Flora	*Dicrodium bande*	Jordan	Low latitude	Tree	5–10	Canopy	590.1		Pinnate		Thick		
	Changhsingian	Cathaysian Flora	*Dicrodium irnense*	Jordan	Low latitude	Tree	5–10	Canopy	4.7		Pinnate		Thick		
	Changhsingian	Cathaysian Flora	*Isoetalean*	Jordan	Low latitude	Herbaceous	0.05–0.1	Ground cover		307	Simple		Thin		
	Changhsingian	Angara-Euromerica flora	*Callipteris zeilleri*	Middle Asia	Middle–high latitude	Tree–shrub		Canopy	96.1		Pinnate to simple net		Thick	Csa	Tree dominant upland forest
	Changhsingian	Euromerica	*conifer*	North China	Middle latitude	Tree		Canopy		20.6	Simple		Thick	Csa	Tree dominant upland forest
	Changhsingian	Euromerica	*Ginkgophyte*	North China	Middle latitude	Tree	20–50	Canopy		500.8	Simple to simple net		Thick		
	Changhsingian	Euromerica	*Germaropteris martinsii*	North China	Middle latitude	Tree to shrub		Canopy	5		Pinnate to simple net		Thick		
	Changhsingian	Gondwana Flora	*Glossopteris*	South Africa	Middle–high latitude	Tree	10–30	Canopy		190.7	Pinnate net		Thin	Dsd	Tree dominant lowland and upland forest
	Changhsingian	Gondwana Flora	*Glossopteris browniana*	South Africa	Middle–high latitude	Tree	10–30	Canopy		2146.1	Pinnate net		Thin		
	Changhsingian	Gondwana Flora	*Glossopteris browniana*	South Africa	Middle–high latitude	Tree	10–30	Canopy		5002.9	Pinnate net	1.24	Thin		
	Changhsingian	Gondwana Flora	*Glossopteris indica*	South Africa	Middle–high latitude	Tree	10–30	Canopy		3635.6	Pinnate net	2.24	Thin		
	Changhsingian	Gondwana Flora	*Sphenophyllum speciosum*	South Africa	Middle–high latitude	Tree to shrub		Understory	113.3		Parallel to simple net	1.83	Thin		

Changhsingian	Gondwana Flora	*Trizygia speciosa*	South Africa	Middle–high latitude	Tree to shrub		Understory	31.2		Parallel to simple net		Thin		
Changhsingian	Gondwana Flora	*Trizygia speciosa*	South Africa	Middle–high latitude	Tree to shrub		Understory	30.8		Parallel to simple net		Thin		
Changhsingian	Cathaysian Flora	*Anshuncladus xinminensis*	South China	Low latitude	Tree	5–80	Canopy		29.5	Simple		Thick	Af	Tree dominant lowland forest with high spatial complex
Changhsingian	Cathaysian Flora	*Anshuncladus aduncatus*	South China	Low latitude	Tree	5–80	Canopy		14.6	Simple		Thick		
Changhsingian	Cathaysian Flora	*Annularia pingloensis*	South China	Low latitude	Tree–shrub	5–10	Understory	1.4		Simple	2.03	Thin		
Changhsingian	Cathaysian Flora	*Annularia shirakii*	South China	Low latitude	Tree–shrub	5–10	Understory	6		Simple	0.94	Thin		
Changhsingian	Cathaysian Flora	*Fascipteris stena*	South China	Low latitude	Tree	5–10	Canopy	221.4		Pinnate	2.55	Thin		
Changhsingian	Cathaysian Flora	*Gigantonoclea acuminatiloba*	South China	Low latitude	Liana		Understory	13802.5		Pinnate net		Thick		
Changhsingian	Cathaysian Flora	*Gigantonoclea guizhouensis*	South China	Low latitude	Liana		Understory	3161.5		Pinnate net		Thick		
Changhsingian	Cathaysian Flora	*Gigantonoclea hallei*	South China	Low latitude	Liana		Understory	6006		Pinnate net	3.10	Thick		
Changhsingian	Cathaysian Flora	*Gigantonoclea longifolia*	South China	Low latitude	Liana		Understory	18545		Pinnate net		Thick		
Changhsingian	Cathaysian Flora	*Gigantonoclea lotaba*	South China	Low latitude	Liana		Understory	17.7		Pinnate net		Thick		
Changhsingian	Cathaysian Flora	*Gigantonoclea rosulata*	South China	Low latitude	Liana		Understory	3435.7		Pinnate net	1.76	Thick		
Changhsingian	Cathaysian Flora	*Gigantopteris cordata*	South China	Low latitude	Liana		Understory	1771		Pinnate net		Thick		
Changhsingian	Cathaysian Flora	*Gigantopteris dictyophylloides*	South China	Low latitude	Liana		Understory	2646.2		Pinnate net		Thick		
Changhsingian	Cathaysian Flora	*Gigantopteris dictyophylloides*	South China	Low latitude	Liana		Understory	7215		Pinnate net		Thick		
Changhsingian	Cathaysian Flora	*Gigantopteris lagrelii*	South China	Low latitude	Liana		Understory	1126.5		Pinnate net	2.16	Thick		
Changhsingian	Cathaysian Flora	*Gigantopteris nicotianaefolia*	South China	Low latitude	Liana		Understory	64169		Pinnate net		Thick		
Changhsingian	Cathaysian Flora	*Lepidodendron acutangulum*	South China	Low latitude	Tree	30–50	Canopy		225	Simple		Thin		
Changhsingian	Cathaysian Flora	*Pecopteris lativenasa*	South China	Low latitude	Tree	5–10	Understory	78.8		Pinnate	5.09	Thin		
Changhsingian	Cathaysian Flora	*Pecopteris marginata*	South China	Low latitude	Tree	5–10	Understory	16.7		Pinnate		Thin		
Changhsingian	Cathaysian Flora	*Rajahia guizhouensis*	South China	Low latitude	Tree		Understory	12.1		Pinnate		Thin		
Changhsingian	Cathaysian Flora	*Schizoneura manchuriensis*	South China	Low latitude	Tree–shrub		Understory	43		Parallel to simple net		Thin		
Changhsingian	Cathaysian Flora	*Sphenophyllum*	South China	Low latitude	Tree		Understory	155.9		Simple		Thin		
Changhsingian	Cathaysian Flora	*Taeniopteris multineris*	South China	Low latitude	Tree		Canopy		7273	Pinnate to simple vet	2.09	Thick		
Changhsingian	Cathaysian Flora	*Tingia gerardii*	South China	Low latitude	Tree	5–10	Canopy	199.6		Simple		Thick		
Changhsingian	Cathaysian-Gondwana Flora	*Sphenopteris taiyuanensis*	Southeast Asia	Low latitude	Tree to shrub		Canopy-underst[illegible]	60.7		Pinnate to simple		Thin	Cfb	Tree dominant lowland and upland forest
Changhsingian	Cathaysian-Gondwana Flora	*Fascipteris stena*	Southeast Asia	Low latitude	Tree to shrub		Understory		180	Pinnate	2.87	Thin		
Changhsingian	Cathaysian-Gondwana Flora	*Lobatannularia multifolia*	Southeast Asia	Low latitude	Tree to shrub	5–10	Understory	22.6		Simple		Thin		
Changhsingian	Cathaysian-Gondwana Flora	*Rajahia guizhouensis*	Southeast Asia	Low latitude	Tree to shrub		Understory	13		Pinnate		Thin		
Changhsingian	Cathaysian-Gondwana Flora	*Taeniopteris*	Southeast Asia	Low latitude	Tree		Canopy		359.8	Pinnate to simple net		Thick		
Changhsingian	Cathaysian-Gondwana Flora	*Glossopteris browniana*	Southeast Asia	Low latitude	Tree	10–30	Canopy		447	Pinnate net		Thin		
PTT to Induan	Survival and pioneer flora in South hemisphere	*Zuberia brownii*	Argentina	Middle–high latitude	Tree to shrub		Canopy	9.4		Pinnate to simple net		Thick	Csa	Tree dominant lowland and upland forest
PTT to Induan	Survival and pioneer flora in South hemisphere	*Zuberia feistmantelii*	Argentina	Middle–high latitude	Tree to shrub		Canopy	5		Pinnate to simple net		Thick		
PTT to Induan	Survival and pioneer flora in South hemisphere	*Dicrodium incisum*	Argentina	Middle–high latitude	Tree to shrub	5–10	Canopy	33.9		Pinnate		Thin		
PTT to Induan	Survival and pioneer flora in South hemisphere	*Cylostrobus sydneyensis*	Australia	High latitude	Herbaceous	0.05–0.1	Ground cover		87	No		Thin	Bsh	Herbaceous lycopod dominant steppe
PTT to Induan	Survival and pioneer flora in South hemisphere	*Isoetes beestonii*	Australia	High latitude	Herbaceous	0.05–0.1	Ground cover		234	Simple		Thin		
PTT to Induan	Survival and pioneer flora in South hemisphere	*Skilliostrobus australis*	Australia	High latitude	Herbaceous	0.05–0.1	Ground cover		417	Simple		Thin		
PTT to Induan	Survival and pioneer flora in South hemisphere	*Tomiostrobus australis*	Australia	High latitude	Herbaceous	0.05–0.1	Ground cover		115	Simple		Thin		
PTT to Induan	Survival and pioneer flora in South hemisphere	*Pleuromeia sternbergii*	Australia	High latitude	Shrub	0.3–2	Canopy		272	Simple		Thin		

Permian Triassic transition (PTT) to Early Triassic	PTT to Induan	Survival and pioneer flora in South hemisphere	*Cylostrobus indicus*	Australia	High latitude	Herbaceous	0.05–0.1	Ground cover		54	No		Thin		
	PTT to Induan	Survival and pioneer flora in North hemisphere	*Pleuromeia rossica*	Europe	Middle latitude	Shrub	0.3–2	Canopy		194.8	Simple		Thin	Bsh	Herbaceous lycopod dominant steppe with survival conifers in the
	PTT to Induan	Survival and pioneer flora in North hemisphere	*Tomiostrobus belozerovii*	Russia	High latitude	Herbaceous	0.05–0.1	Ground cover		79	Simple		Thin	Bsh	Herbaceous lycopod dominant steppe with survivals in the lowland and upland
	PTT to Induan	Survival and pioneer flora in North hemisphere	*Tomiostrobus bulbosus*	Russia	High latitude	Herbaceous	0.05–0.1	Ground cover		59	Simple		Thin		
	PTT to Induan	Survival and pioneer flora in North hemisphere	*Tomiostrobus convexus*	Russia	High latitude	Herbaceous	0.05–0.1	Ground cover		72	Simple		Thin		
	PTT to Induan	Survival and pioneer flora in North hemisphere	*Tomiostrobus gorskyii*	Russia	High latitude	Herbaceous	0.05–0.1	Ground cover		167	Simple		Thin		
	PTT to Induan	Survival and pioneer flora in North hemisphere	*Tomiostrobus radiatus*	Russia	High latitude	Herbaceous	0.05–0.1	Ground cover		154	Simple		Thin		
	PTT to Induan	Survival and pioneer flora in North hemisphere	*Isoetes innae*	Russia	High latitude	Herbaceous	0.05–0.1	Ground cover		6	Simple		Thin		
	PTT to Induan	Survival and pioneer flora in North hemisphere	*Pleuromeia rossica*	Russia	High latitude	Shrub	0.3–2	Canopy		151	Simple		Thin		
	PTT to Induan	Survival and pioneer flora in North hemisphere	*Tomiostrobus migayi*	Russia	High latitude	Herbaceous	0.05–0.1	Ground cover		189	Simple		Thin		
	PTT to Induan	Survival and pioneer flora in North hemisphere	*Tomiostrobus radiatus*	Russia	High latitude	Herbaceous	0.05–0.1	Ground cover		287	Simple		Thin		
	PTT to Induan	Survival and pioneer flora in North hemisphere	*Pecopteris*	South China	Low latitude	Tree to shrub	5–10	Understory	30.5		Pinnate		Thin	Cfa	Herbaceous lycopod dominant steppe with few survival points in lowland and refuges in upland
	PTT to Induan	Survival and pioneer flora in North hemisphere	*Germaropteris martinsii*	South China	Low latitude	Tree to shrub		Canopy	1.4		Pinnate to simple net		Thick		
	PTT to Induan	Survival and pioneer flora in North hemisphere	*Tomiostrobus angusta*	South China	Low latitude	Herbaceous	0.05–0.1	Ground cover		75	Simple	0.14	Thin		
	PTT to Induan	Survival and pioneer flora in North hemisphere	*Tomiostrobus brevicystis*	South China	Low latitude	Herbaceous	0.05–0.1	Ground cover		293	Simple		Thin		
	PTT to Induan	Survival and pioneer flora in North hemisphere	*Tomiostrobus zeilleri*	South China	Low latitude	Herbaceous	0.05–0.1	Ground cover		150	Simple	0.08	Thin		
Early Triassic	Olenekian	Pioneer and recovery flora in North hemisphere	*Pleuromeia*	North China	Middle-high latitude	Shrub	0.3–2	Canopy		363.2	Simple		Thin	Cfa	Herbaceous lycopod dominant steppe with few refuges in upland
	Olenekian	Pioneer and recovery flora in North hemisphere	*Anomopteris*	North China	Middle-high latitude	Tree to shrub		Canopy-underst	13.5		Pinnate to simple net		Thin		
	Olenekian	Pioneer and recovery flora in North hemisphere	*Neuropteridium marginatum*	South China	Low latitude	Tree		Canopy	45.8		Pinnate net		Thin	Csa	Tree dominant upland forest
	Olenekian	Pioneer and recovery flora in North hemisphere	*Albertia latifolia*	South China	Low latitude	Tree		Canopy		95.3	Parallel	3.04	Thick		
	Olenekian	Pioneer and recovery flora in North hemisphere	*Albertia elliptica*	South China	Low latitude	Tree		Canopy		24.5	Parallel		Thick		
	Olenekian	Pioneer and recovery flora in North hemisphere	*Voltzia*	South China	Low latitude	Tree	0.5–2	Canopy		87.8	Simple	0.22	Thick		
	Anisian	Recovery flora in South hemisphere	*Dicrodium odontopteroides*	Antarctic	High latitude	Tree	5–10	Canopy	46.9		Pinnate	1.71	Thin	Dfa	Tree dominant lowland and upland forest
	Anisian	Recovery flora in South hemisphere	*Dicrodium crassinervis*	Antarctic	High latitude	Tree	5–10	Canopy	33.8		Pinnate		Thin		
	Anisian	Recovery flora in South hemisphere	*Dicrodium coruaceum*	Antarctic	High latitude	Tree	5–10	Canopy	778.6		Pinnate	1.20	Thin		
	Anisian	Recovery flora in South hemisphere	*Dicrodium fremounvensis*	Antarctic	High latitude	Tree	5–10	Canopy	58.8		Pinnate	1.18	Thin		
	Anisian	Recovery flora in South hemisphere	*Dicrodium dubium*	Antarctic	High latitude	Tree	5–10	Canopy	208.8		Pinnate		Thin		
	Anisian	Recovery flora in South hemisphere	*Dicrodium*	Antarctic	High latitude	Tree	5–10	Canopy	19.7		Pinnate		Thin		
	Anisian	Recovery flora in South hemisphere	*Fraxinopsis andiun*	Argentina	High latitude	Tree		Canopy		179.3	Simple	0.74	Thick		Tree dominant upland and

Middle Triassic	Anisian	Recovery flora in South hemisphere	*Scytophyllum*	Argentina	High latitude	Tree-shrub		Canopy	16.3		Pinnate		Thick	Csb	Tree dominant upland and lowland forest with herbaceous lycopod steppe
	Anisian	Recovery flora in South hemisphere	*Lepacyclotes*	Argentina	High latitude	Herbaceous	0.1–0.3	Ground cover		46.1	Simple		Thin		
	Anisian	Recovery flora in North hemisphere	*Lepacyclotes zeilleri*	Europe	Middle latitude	Herbaceous	0.1–0.3	Ground cover		225	Simple	0.02	Thin	Am	Tree dominant upland and lowland forest with herbaceous lycopod steppe
	Anisian	Recovery flora in North hemisphere	*Lepacyclotes kiechneri*	Europe	Middle latitude	Herbaceous	0.1–0.3	Ground cover		97	Simple		Thin		
	Anisian	Recovery flora in North hemisphere	*Sigillcampeia blaui*	Europe	Middle latitude	Shrub		Canopy		1335	No		Thin		
	Anisian	Recovery flora in North hemisphere	*Lepacyclotes zeilleri*	North China	Middle latitude	Herbaceous	0.1–0.3	Ground cover		96.4	Simple	0.03	Thin	Cfa	Tree dominant upland and lowland forest with herbaceous lycopod steppe
	Anisian	Recovery flora in North hemisphere	*Lepacyclotes ermayinensis*	North China	Middle latitude	Herbaceous	0.1–0.3	Ground cover		272	Simple		Thin		
	Anisian	Recovery flora in North hemisphere	*Lepacyclotes ordosensis*	North China	Middle latitude	Herbaceous	0.1–0.3	Ground cover		100.5	Simple	0.02	Thin		
	Anisian	Recovery flora in North hemisphere	*Pleuromeia rossica*	North China	Middle latitude	Shrub	0.3–2	Canopy		168	Simple		Thin		
	Anisian	Recovery flora in North hemisphere	*Pleuromeia sternbergii*	North China	Middle latitude	Shrub	0.3–2	Canopy		1269	Simple		Thin		
	Anisian	Recovery flora in North hemisphere	*Voltzia*	South China	Low latitude	Tree	0.5–2	Canopy		20.3	Simple	0.43	Thick	Am	Tree dominant upland and lowland forest with herbaceous lycopod steppe
	Anisian	Recovery flora in North hemisphere	*Todites shensiensis*	South China	Low latitude	Shrub-herbaceo	0.5–1	Understory	18		Pinnate net		Thin		
	Anisian	Recovery flora in North hemisphere	*Pelourdea* (*Yuccites*) *vogesiacuus*	South China	Low latitude	Tree	5–10	Canopy		9429	Simple	0.88	Thick		
	Anisian	Recovery flora in North hemisphere	*Scytophyllum*	South China	Low latitude	Tree-shrub		Canopy	20.5		Pinnate		Thick		
	Anisian	Recovery flora in North hemisphere	*Lepacyclotes latiloba*	South China	Low latitude	Herbaceous	0.1–0.3	Ground cover		636	Simple		Thin		
	Anisian	Recovery flora in North hemisphere	*Lepacyclotes sangzhiensis*	South China	Low latitude	Herbaceous	0.1–0.3	Ground cover		125	Simple		Thin		
	Anisian	Recovery flora in North hemisphere	*Lepacyclotes brevicystis*	South China	Low latitude	Herbaceous	0.1–0.3	Ground cover		2012	Simple		Thin		
	Anisian	Recovery flora in North hemisphere	*Lepacyclotes zeilleri*	South China	Low latitude	Herbaceous	0.1–0.3	Ground cover		660	Simple	0.02	Thin		
	Anisian	Recovery flora in North hemisphere	*Pleuromeia hunanensis*	South China	Low latitude	Shrub	0.3–2	Canopy		1188	Simple		Thin		
	Anisian	Recovery flora in North hemisphere	*Pleuromeia marginulata*	South China	Low latitude	Shrub	0.3–2	Canopy		1040	Simple		Thin		
	Anisian	Recovery flora in North hemisphere	*Lepacyclotes circularis*	USA	Middle latitude	Herbaceous	0.1–0.3	Ground cover		637	Simple		Thin	Am	Upland tree dominant forest with herbaceous lycopod

* Pinna size, for compound leaf only measure the biggest pinna, for simple leaf measure the whole leaf, petiole is exclueded
* Vein density is defined as the vein length per area which is measured with ImageJ
* Cuticle thickness is relative semi-estimation. Further measurement should be done in the future
* Explaination of the climate zone is in Table S6 in the next sheet

Table S6. Flora cliamatical classification for choosing recent plant functional groups for the fossil floras.

	Equatorial			Arid				Temperate				Boreal					Tundra
	A			B				C				D					E
Abbrev.	**Af**	**Am**	**Aw**	**Bwh**	**Bwk**	**Bsh**	**Bsk**	**Cfa**	**Cfb**	**Csa**	**Csb**	**Dfa**	**Dfb**	**Dsb**	**Dsc**	**Dsd**	**E**
Feature	Tropical	Tropical	Tropical	Arid	Arid	Arid	Arid	Temperate	Temperate	Temperate	Temperate	Continental	Continental	Continental	Continental	Continental	Tundra
	Rainforest	Monsoon	Savanna	Desert	Desert	Steppe	Steppe	No dry season	No dry season	Dry summer	Dry summer	No dry season	No dry season	Dry summer	Dry summer	Dry summer	
				Hot	Cold	Hot	Cold	Hot summer	Warm summer	Hot summer	Warm summer	Hot summer	Warm summer	Warm summer	Cold summer	Very cold summer	
NPPL	-1		-18	-20	-16			-3	-4	-17	-7	-8	-10	-11	-12	-9	
Changhsingian	South China		United States					China Xizang	Turkey	North China	China Xinjiang	Russia high latitude area	Australia	Argentina	Antarctica	South Africa	
			Italy					Laos	Thailand	Russia low latitude area			Indonesia		India		
			Austria														
			Germany														
			United Kingdom														
NPPL						-19		-13	-7	-17	-15						
Induan						Australia		North China	Russia high latitude area	Argentina	South Africa						
						Spain		South China	Norway	Greenland	Germany						
						Mongolia				Ireland	France						
						Kazakstan				Poland	Serbia						
						Russia low latitude area				Canada							
						China Xinjiang											
						India											
NPPL			-18			-19		-6	-7	-17	-15				-12	-11	
Olenekian			Austria			Greenland		Russia high latitude area	South Africa	South China	Spain				Aruba	India ?	
			Norway			Tajikstan		Germany		France	United Kingdom						
			Italy			Kazakstan		North China		Russia low latitude area	Italy						
			Madagascar			Japan				Hungary							
										Australia							
NPPL		-2	-18			-19		-5	-6	-17	-7	-8					
		France	Poland			Tajikstan		China Xinjiang	Syria	Canada	Russia low latitude area	Antarctica					

Anisian		South China	United States			Switzerland		North China	Brazil	United Kingdom	India	Aruba					
		Germany	Turkey					Australia	New Zealand	Spain	Argentina	Russia low latitude area					
		Italy	Italy								South Africa						
											Mongolia						

* (1)–(20) represents the corresponding nearest living flora, see details in Table S7 in the next sheet

Table S7. Simplified information of nearest living flora (location, NPPL and character from ref. 130 in table references; Mean annual temperature and precipitation from https://en.wikipedia.org/wiki/List_of_cities_by_average_temperature).

NO.	Location	NPPL (g C/m^2/y)	Longitude	Latitude	Character	Mean annual temperature (℃)	Mean annual precipitation (mm)
(1)+	Thailand-Nakhon Ratchasima	1680	99.8	7.58	Tropical (evergreen)-island	27.3	1200–4500
(1)-	Malaysia	1495	102.31	2.98	Tropical (evergreen)-island	27.3	3085
(2)+	Colombia	1636	-73.56	6.39	Tropical (evergreen)-continental	24	3700
(2)-	Brazil	1152	-69.75	-5.75	Tropical (evergreen)-continental	28	1500–3000
(3)+	China	1310	95.1	30.15	Subtropical (deciduous)	22	580–2700
(3)-	Papua New Guine	1042	145.18	-6	Tropical (evergreen)	26.9	2500
(4)+	Germany	1240	9.5	49	Temperate (deciduous)	13	500-2000
(4)-	New Zealand	1120	176	-39	Temperate–Subtropical (evergreen)	17	600–1500
(5)+	USA-Washington	1069	-122.61	44.68	Temperate (coniferous)	19	890
(5)-	Jamaica	981	-76.65	18.08	Tropical (evergreen)	31	604
(6)+	USA-Tennessee	827	-84.29	35.96	Temperate (deciduous)	16	1300
(6)-	China-Henan	820	111.57	34.9	Temperate (deciduous)	16	500–900
(7)+	Belgium	710	5	49.75	Temperate (deciduous)	13	795
(7)-	China-Daxinanlin	688	123	49	Temperate (deciduous)	-2.8	530–700
(8)+	Siberia-Верхоянкий район	931	83	58	Boreal (evergreen)	-0.6	435
(8)-	Siberia	911	103	53	Boreal (evergreen)	5	420
(9)+	USA-Alaska	612	-148.25	64.75	Boreal (deciduous)	-2.3	300
(9)-	Russia	585	34	62	Boreal (evergreen)	0.3	530
(10)+	Russia-Суоярвсий район	590	34	62	Boreal (evergreen)	0.3	530
(10)-	Canada	565	-115.5	50.2	Boreal (evergreen)	4.4	400
(11)+	Sweden	410	16.5	60.82	Boreal (coniferous)	6.6	542
(11)-	Canada	394	-106.2	53.63	Boreal (deciduous)	4.4	400
(12)+	Canada	370	-98.7	55.9	Boreal–temperate (deciduous)	3	1000
(12)-	Finland	368	29.32	66.37	Boreal (evergreen)	2.7	400–650
(13)+	Australia	380	119.25	-21.25	Shrub	34	360
(13)-	China-Yunnan	358	101.25	23.75	Subtropical forest	18	375–2400
(14)+	Argentina	320	-64.25	-25.75	Xeric	20.4	240
(14)-	Venezuela	310	-67.42	8.93	Tropical island Savanna	25.9	1290
(15)+	France	260	2.7	48.4	Temperate (deciduous)	12.5	655
(15)-	Ivory Coast	250	-5.03	6.22	Humid savanna	26	1159
(16)+	Kazakhstan	170	71	51.6	Cold desert steppe (C3 grassland)	11	200–500
(16)-	Russia-Респу́блка Тыва́	137	94.42	51.83	Cold desert steppe (C3 grassland)	-8	180–300
(17)+	India	740	85.1	26.42	Temperate (dry deciduous)	27.1	1220
(17)-	Argentina	719	-59.75	-26.75	Xeric	31	300
(18)+	Kenya	162	36.83	-1.33	Savanna (grassland)	29.3	250
(18)-	Australia	111	146.27	-26.4	Savanna (C3 grassland)	27.6	419
(19)+	Syria	110	38	35	Steppe (C3 grassland)	24	252
(19)-	USA-New Mexico	93	-106.85	32.6	Sub-tropical semi-desert	18	380
(20)+	Algeria	80	3	33	Dessert	28.3	330
(20)-	Australia	40	146.22	-26.42	Dessert–shrub	27.6	300

* ‘+’ is the upper limit of the NPP range; ‘-’ is the lower limit of the NPP range.

122. Yin, H. & Tong, J. Multidisciplinary high-resolution correlation of the Permian–Triassic boundary. *Palaeogeogr Palaeoclimatol Palaeoecol* 143, 199–212 (1998).

123. Xie, S. *et al.* Changes in the global carbon cycle occurred as two episodes during the Permian–Triassic crisis. *Geology* 35, 1083 (2007).

124. Xie, S., Hong, F., Cao, C., Wang, J. C. & Lai, X. Episodic changes of the earth surface system across the Permian–Triassic boundary: molecular geobiological records. *Acta Palaeontological Sin* 3, 496–506 (2009).

125. Shen, S. *et al.* Calibrating the End-Permian Mass Extinction. *Science* 334, 1367–1372 (2011).

126. Song, H., Wignall, P. B. & Dunhill, A. M. Decoupled taxonomic and ecological recoveries from the Permo-Triassic extinction. *Sci Adv* 4, (2018).

127. Grasby, S. E. *et al.* Isotopic signatures of mercury contamination in latest Permian oceans. *Geology* 45, 55–58 (2017).

128. Shen, J. *et al.* Evidence for a prolonged Permian–Triassic extinction interval from global marine mercury records. *Nat Commun* 10, 1563 (2019).

129. Fontaine, H. Permian of Southeast Asia: an overview. *J Asian Earth Sci* 20, 567–588 (2002).

130. Shi, G. R. & Shen, S. A Changhsingian (Late Permian) brachiopod fauna from Son La, northwest Vietnam. *J Asian Earth Sci* 16, 501–511 (1998).

131. Bercovici, A. *et al.* Permian continental paleoenvironments in Southeastern Asia: New insights from the Luang Prabang Basin (Laos). *J Asian Earth Sci* 60, 197–211 (2012).

132. Archangelsky, S. & Wagner, R. H. Glossopteris anatolica sp. nov. from uppermost Permian strata in southeast Turkey. *Geology* 37, 81–91 (1983).

133. Powell, J. H. *et al.* The Permian-Triassic boundary, Dead Sea, Jordan; transitional alluvial to marine depositional sequences and biostratigraphy. *Riv. Ital. Paleontol. S.* 122, 23–39 (2016).

134. Retallack, G. J., Smith, R. M. H. & Ward, P. D. Vertebrate extinction across Permian–Triassic boundary in Karoo Basin, South Africa. *Geol Soc Am Bull* 115, 1133 (2003).

135. Gastaldo, R. A., Neveling, J., Geissman, J. W. & Kamo, S. L. A lithostratigraphic and magnetostratigraphic framework in a geochronologic context for a purported Permian–Triassic boundary section at Old (West) Lootsberg Pass, Karoo Basin, South Africa. *GSA Bulletin* 130, 1411–1438 (2018).

136. Gastaldo, R. A. *et al.* Is the vertebrate-defined Permian-Triassic boundary in the Karoo Basin, South Africa, the terrestrial expression of the end-Permian marine event? *Geology* 43, 939–942 (2015).

137. Gastaldo, R. A. *et al.* The base of the *Lystrosaurus* Assemblage Zone, Karoo Basin, predates the end-Permian marine extinction. *Nat Commun* 11, 1428 (2020).

138. Mundil, R., Metcalfe, I., Chang, S. & Renne, P. R. The Permian-Triassic boundary in Australia: New radio-isotopic ages. *Geochim Cosmochim Acta* 70, A436 (2006).

139. Metcalfe, I., Crowley, J. L., Nicoll, R. S. & Schmitz, M. High-precision U-Pb CA-TIMS calibration of Middle Permian to Lower Triassic sequences, mass extinction and extreme climate-change in eastern Australian Gondwana. *Gondwana Res* 28, 61–81 (2015).

140. Laurie, J. R. *et al.* Calibrating the middle and late Permian palynostratigraphy of Australia to the geologic time-scale via U–Pb zircon CA-IDTIMS dating. *Australian J Earth Sci* 63, 701–730 (2016).

141. Fielding, C. R. *et al.* Age and pattern of the southern high-latitude continental end-Permian extinction constrained by multiproxy analysis. *Nat Commun* 10, 385 (2019).

142. Retallack, G. J. & Krull, E. S. Landscape ecological shift at the Permian-Triassic boundary in Antarctica. *Australian J Earth Sci* 46, 785–812 (1999).

143. Retallack, G. J. Earliest Triassic Claystone Breccias and Soil-Erosion Crisis. *J Sediment Res* 75, 679–695 (2005).

144. Sato, A. M., Llambías, E. J., Basei, M. A. S. & Castro, C. E. Three stages in the Late Paleozoic to Triassic magmatism of southwestern Gondwana, and the relationships with the volcanogenic events in coeval basins. *J South Am Earth Sci* 63, 48–69 (2015).

145. Mays, C. *et al.* Refined Permian–Triassic floristic timeline reveals early collapse and delayed recovery of south polar terrestrial ecosystems. *GSA Bulletin* 132, 1489–1513 (2020).

146. Guo, W. *et al.* Late Permian–Middle Triassic magnetostratigraphy in North China and its implications for terrestrial-marine correlations. *Earth Planet Sci Lett* 585, 117519 (2022).

147. Liu, J. & Abdala, N. F. Therocephalian (Therapsida) and chroniosuchian (Reptiliomorpha) from the Permo-Triassic transitional Guodikeng Formation of the Dalongkou Section, Jimsar, Xinjiang, China. *Vertebrata Palasiatica* 1000–3118 (2017).

148. Cao, C., Wang, W., Liu, L., Shen, S. & Summons, R. E. Two episodes of ^{13}C-depletion in organic carbon in the latest Permian: Evidence from the terrestrial sequences in northern Xinjiang, China. *Earth Planet Sci Lett* 270, 251–257 (2008).

149. Bourquin, S., Durand, M., Diez, J. B., Broutin, J. & Fluteau, F. The Permian-Triassic boundary and Early Triassic sedimentation in western European basins; an overview. *J. Iber. Geol.* 33, 221–236 (2007).

150. Dai, X. *et al.* A Mesozoic fossil lagerstätte from 250.8 million years ago shows a modern-type marine ecosystem. *Science* 379, 567–572 (2023).

151. Davydov, V. I. & Karasev, E. V. The influence of the Permian-Triassic magmatism in the Tunguska Basin, Siberia on the regional floristic biota of the Permian-Triassic Transition in the region. *Front Earth Sci (Lausanne)* 9, (2021).

152. Shi, X., Zhang, W., Yu, J. X., Chu, D. L. & Huang, C. The flora from Karamay Formation in the south and north of Tianshan Mountain, Xinjiang. *Geol Sci Technol Info* 1, 55–61 (2014).

153. Cai, Y. *et al.* Wildfires and deforestation during the Permian–Triassic transition in the southern Junggar Basin, Northwest China. *Earth Sci Rev* 218, 103670 (2021).

154. Mickelson, D. L., Huntoon, J. E. & Kvale, E. P. The diversity and stratigraphic distribution of pre-dinosaurian communities from the Triassic Moenkopi Formation. *New Mexico Museum Nat Hist Sci Bull* 34, 132–137 (2006).

155. Shu, W. *et al.* Permian–Middle Triassic floral succession in North China and implications for the great transition of continental ecosystems. *Bull Geol Soc Am* 135, 1747–1767 (2023).

156. Bercovici, A., Cui, Y., Forel, M.-B., Yu, J. & Vajda, V. Terrestrial paleoenvironment characterization across the Permian–Triassic boundary in South China. *J Asian Earth Sci* 98, 225–246 (2015).

157. Ran, W. J. Taxonomy and morphological evolution of the isoetalean lycopsids during Induan - Anisian in Hunan, Guizhou and Yunnan, South China. (China University of Geosciences (Wuhan)., Wuhan, 2021).

158. Xue, Q. The evolution of fossil plants during Late Permian and Early Triassic in Western Guizhou and Eastern Yunnan, South China. (China University of Geosciences (Wuhan)., Wuhan, 2019).

159. Li, H., Yu, J., McElwain, J. C., Yiotis, C. & Chen, Z.-Q. Reconstruction of atmospheric CO_2 concentration during the late Changhsingian based on fossil conifers from the Dalong Formation in South China. *Palaeogeogr Palaeoclimatol Palaeoecol* 519, 37–48 (2019).

160. Meng, F. S. Floral palaeoecological environment of the Badong Formation in the Yangtze gorges area. *Geol Min Resour South China* 1–13 (1996).

161. Barbolini, N., Bamford, M. K. & Rubidge, B. Radiometric dating demonstrates that Permian spore-pollen zones of Australia and South Africa are diachronous. *Gondwana Res* 37, 241–251 (2016).

162. Todd, C. N., Roberts, E. M. & Charles, A. J. A revised Permian–Triassic stratigraphic framework for the northeastern Galilee Basin, Queensland, Australia, and definition of a new Middle–Upper Triassic sedimentary unit. *Australian J Earth Sci* 69, 113–134 (2022).

163. Evans, T. *et al. Context Statement for the Galilee Subregion. Product 1.1 from the Lake Eyre Basin Bioregional Assessment*. (2014).

164. Farabee, M. J., Taylor, E. L. & Taylor, T. N. Correlation of permian and triassic palynomorph assemblages from the central Transantarctic Mountains, Antarctica. *Rev Palaeobot Palynol* 65, 257–265 (1990).

165. Hills, A. *et al. Late Permian and Triassic Palynomorphs from the Allan Hills, Central Transantarctic Mountains, South Victoria Land, Antarctica*. vol. 106 https://about.jstor.org/terms (2014).

166. Lindström, S. & McLoughlin, S. Synchronous palynofloristic extinction and recovery after the end-Permian event in the Prince Charles Mountains, Antarctica: Implications for palynofloristic turnover across Gondwana. *Rev Palaeobot Palynol* 145, 89–122 (2007).

167. Elliot, D. H. & Fanning, C. M. Detrital zircons from upper Permian and lower Triassic Victoria Group sandstones, Shackleton Glacier region, Antarctica: Evidence for multiple sources along the Gondwana plate margin. *Gondwana Res* 13, 259–274 (2008).

168. Elliot, D. H. & Grimes, C. G. Triassic and Jurassic strata at Coombs Hills, south Victoria Land: stratigraphy, petrology and cross-cutting breccia pipes. *Antarct Sci* 23, 268–280 (2011).
